# Selective Noble Gas Inclusion in Pentagon-Dodecahedral X_20_-Cages †

**DOI:** 10.3390/molecules28155676

**Published:** 2023-07-27

**Authors:** Christopher Weinert, Dušan Ćoćić, Ralph Puchta, Rudi van Eldik

**Affiliations:** 1 Fakultät Angewandte Mathematik, Physik und Allgemeinwissenschaften, Technische Hochschule Nuremberg Georg Simon Ohm, Keßlerplatz 12, 90489 Nuremberg, Germany; 2 Department of Chemistry, Faculty of Science, University of Kragujevac, Radoja Domanovića 12, P.O. Box 60, 34000 Kragujevac, Serbia; 3 Inorganic Chemistry, Department of Chemistry and Pharmacy, University of Erlangen-Nuremberg, Egerlandstr. 1, 91058 Erlangen, Germany; 4 Central Institute for Scientific Computing (CISC), University of Erlangen-Nuremberg, Martensstr. 5a, 91058 Erlangen, Germany; 5 Computer Chemistry Center, Department of Chemistry and Pharmacy, University of Erlangen-Nuremberg, Nägelsbachstr. 25, 91052 Erlangen, Germany; 6 Faculty of Chemistry, Nicolaus Copernicus University in Toruń, Gagarina 7, 87-100 Toruń, Poland

**Keywords:** noble gases, dodecahedrane, host-guest chemistry, DFT

## Abstract

Using DFT-based computational chemistry calculations (ωB97XD/def2-tzvp//ωB97XD/def2-svp/svpfit + ZPE(ωB97XD/def2-svp/svpfit)), binding energies of noble gases encapsulated in a series of dodecahedrane molecules (general formula: X_20_H_20_ where X = C, Si, Ge, Sn and Pb, and X_20_ where X = N, P, As, Sb and Bi) were calculated to learn about the noble gas selectivity. Based on calculated binding energies, the Sn_20_H_20_ cage can best accommodate noble gases with a medium size radius (Ar and Kr), while the Pb_20_H_20_ dodecahedrane cage is best suited for noble gases with the larger radii (Xe and Rn). On the other hand, from the elements of the V main group of the periodic table, the Bi_20_ cage has shown the best results to selectively encapsulate Ar and Kr, with the amounts of energy being released being −5.24 kcal/mol and −6.13 kcal/mol, respectively. By monitoring the geometric changes of all here-reported host cages upon encapsulating the noble gas guest, the host has shown minor to no flexibility, testifying to the high rigidity of the dodecahedrane structure which was further reflected in very high encapsulating energies.

## 1. Introduction

**Scheme 1:**
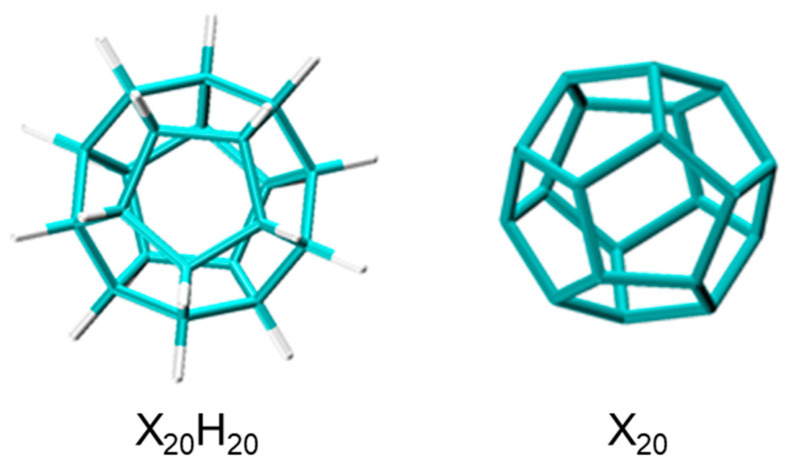
General structural representation of X_20_H_20_ and X_20_ investigated hosts.

Discovery of the C_60_ molecule, better known as fullerene [1], attracted much attention to further investigate the icosahedral molecules. The icosahedral dodecahedrane with its *I_h_* symmetry, C_20_H_20_ (see Scheme 1) was synthesized, and its IR and Raman active frequencies were reported in 1982 [2,3], later on followed by X-ray structural analysis [4]. Structural and stability investigations for a series of dodecahedrane molecules followed [5,6,7,8,9], which suggested that the interior diameter of dodecahedranes (of about ~5 Å) can be exploited for accommodating smaller-size guest species. Special attention was given to the potential applications of icosahedral dodecahedranes as host molecules for encapsulating noble gas molecules, i.e., to act as a preferential absorbent of a hydrophobic guests with applications in purification [10], separation [11], storage [12], catalysis [13,14], and intact transportation [15] of a target solute. Prinzbach et al. [16] have successfully “shot” a helium atom into C_20_H_20_ by using an experimental procedure developed for fullerenes [17]. This encapsulated species is fascinating because the steric compression within the cavity is severe and the barrier to penetrating intact C_20_H_20_ must be very high. Nevertheless, investigating encapsulating properties of small species by dodecahedrane molecules (see Scheme 1) remains experimental and still very challenging.

Therefore, approaching this field by applying computational chemistry can give a wider picture on the capability and tendency of dodecahedrane molecules as hosts for small molecules [18,19]. The resulting supramolecular structures reminds one of the well-known hydrogen model according to Niels Bohr with one proton (here noble gas atom) in the center and a shell around. 

In this article, the focus is on investigating the selective encapsulation capabilities of noble gases by molecules of dodecahedrane structures which molecular skeleton is formed by atoms of the group IV (general formula X_20_H_20_, where X = C, Si, Ge, Sn and Pb) and group V (general formula X_20_, where X = N, P, As, Sb and Bi) of the periodic table.

## 2. Computational Details

All computational calculations were performed using the ωB97XD [20] theory level. Structures of the investigated systems were optimized by applying the def2-svp/[21,22] svpfit [23] basis set (optimized coordinates of the structures reported here are given in Table S1 ESI) with calculations of the vibration frequencies at the same theory level. We selected dispersion corrected DFT to overcome the well-documented shortcomings of MP2 based methods. For obvious reasons the systems were too big for reliable Coupled Cluster calculations. [21,22] The obtained structures were characterized as minima, transition states or saddle points of higher order by examining the vibrational frequencies (number of imaginary frequencies for the systems reported in the manuscript are listed in Tables S2–S4 ESI) together with the BSSE energies. Suitability of the used theory level has been reported elsewhere [24,25,26,27,28]. For comparison reasons, encapsulation capabilities of cages Pb_20_H_20_ and Bi_20_ were examined using APFD [29] functional and B3LYP [30,31,32] functional in combination with Grimme’s dispersion correction with Becke–Johnson Damping [33] in both cases with def2-svp/svpfit functional for structure optimization. Afterwards, single point calculations at the ωB97XD/def2-svp/svpfit structures were performed at the ωB97XD/def2-tzvp [34] theory level, the energies of which have further been used in discussing the encapsulation affinities of the investigated systems, corrected to zero-point vibration energies from ωB97XD/def2-svp/svpfit calculations (ωB97XD/def2-tzvp//ωB97XD/def2-svp/svpfit + ZPE(ωB97XD/def2-svp/svpfit). The same procedure was performed in a case of two other sample theory levels (APFD and B3LYP-GD3BJ). The GAUSSIAN suite of programs was used with the input templates provided in Table S5 ESI [35]. Non-covalent interactions (NCI) [36] taking place between the dodecahedrane cage hosts and the noble gases were investigated using the Multiwfn program (http://sobereva.com/multiwfn/ (accessed on 20 July 2023)) [37] at the ωB97XD/def2tzvp theory level.

## 3. Results and Discussion

The investigation of favorable selective complexation properties can be defined by two criteria, viz. comparison of the geometric changes of the host upon encapsulation the guest and an appropriated reaction energy, 23^c^ [26,38,39]. For the purpose of monitoring the energy change of an encapsulation process, constructing a model reaction (1) as follows is a most suitable approach, where Ng represents a noble gas and the host is a selected dodecahedrane:

Ng + Host → [Ng ⊂ Host] (1)

The results of the computed complexation energies for dodecahedranes of a general formula X_20_H_20_ are reported in Table 1, whereas for dodecahedrane X_20_ in Table 2, they are presented with regard to the noble gas radii [40].

**Figure 1:**
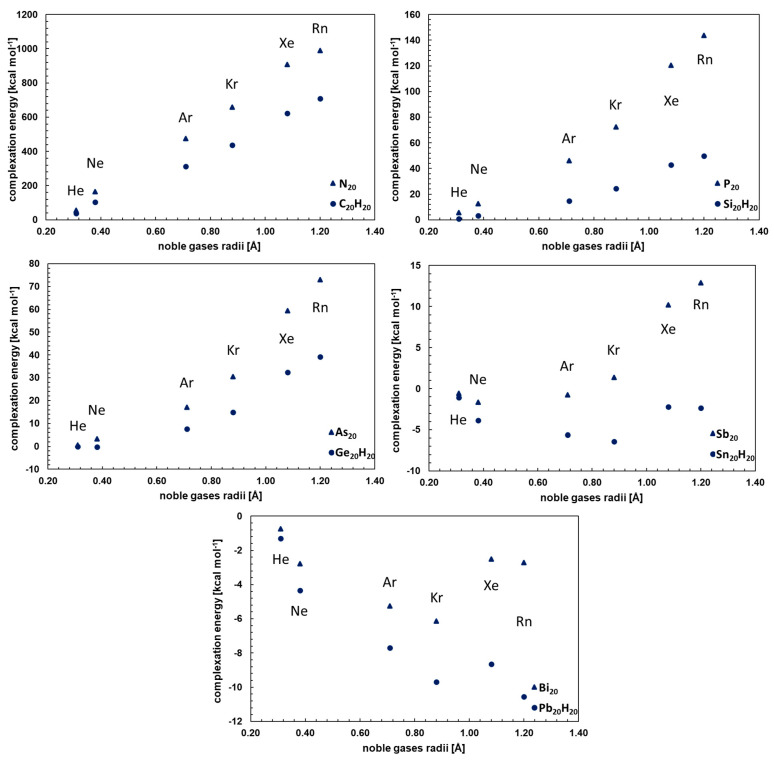
Calculated complexation energies (E_com_) of X_20_ and X_20_H_20_ plotted against the noble gas radii.

As can be seen in Table 1, carbon-based dodecahedranes exhibit very large (unreasonable) amounts of encapsulation energy ranging from 36.94–709.69 kcal/mol. Going down the IV main group along the periodic table, the silicon dodecahedrane cage has a significantly lower encapsulation energy for noble gases, which are linearly increasing with the increasing size of the noble gases radii (Figure 1). The germanium-based dodecahedrane cage has encapsulation energy for He and Ne of ~0 kcal/mol, with a further linear increase in the encapsulation energy going from Ar to Rn (from 7.64 kcal/mol to 39.28 kcal/mol, respectively). The tin cage releases energy upon hosting all noble gases, with the largest amount of energy released for hosting Ar and Kr (−5.63 kcal/mol and −6.41 kcal/mol, respectively), where He and Ne can be considered too small and Xe and Rn too large for the selected Sn_20_H_20_ cavity size. Noble gases with the smaller radii (He and Ne) are rather small for the tin cage cavity, while the ones with the larger radii (Xe and Rn) exhibit lower energy released due to their volume. The lead dodecahedrane cage better accommodates noble gases with larger atomic radii (Figure 1), ranging from −1.31 kcal/mol for He to the largest amount of energy released for Rn (−10.55 kcal/mol).

In Table 2, complexation (encapsulation) energies are presented for the dodecahedrane cages based on elements of the V main group of the periodic table. The nitrogen-based cage exhibits very high encapsulation energies, going from 57.18 kcal/mol to 989.41 kcal/mol for the largest noble gas Rn. The phosphorus cage accommodates noble gases with significantly lower encapsulation energies, but still unreasonably large amounts for the noble gases with larger radii (Kr, Xe and Rn). The arsenic-based dodecahedrane cage has encapsulation energy for He of ~0 kcal/mol, with a further linear increase (Figure 1) of the encapsulation energy going from Ne to Rn (from 3.34 kcal/mol to 73.03 kcal/mol, respectively). In the case of antimony cage noble gases, He, Ne, Ar and Kr have an error margin of ~0 kcal/mol, while Xe and Rn have encapsulation energies of 10.22 kcal/mol and 12.90 kcal/mol, respectively. The bismuth cage, like the previously described tin cage, releases energy upon hosting all noble gases, with the largest amount of energy released for hosting the noble gases Ar and Kr (−5.24 kcal/mol and −6.13 kcal/mol, respectively). Noble gases with the smaller radii (He and Ne) are rather small for the bismuth cage cavity, while the ones with the larger radii (Xe and Rn) exhibit lower energy released due to their volume.

**Figure 2:**
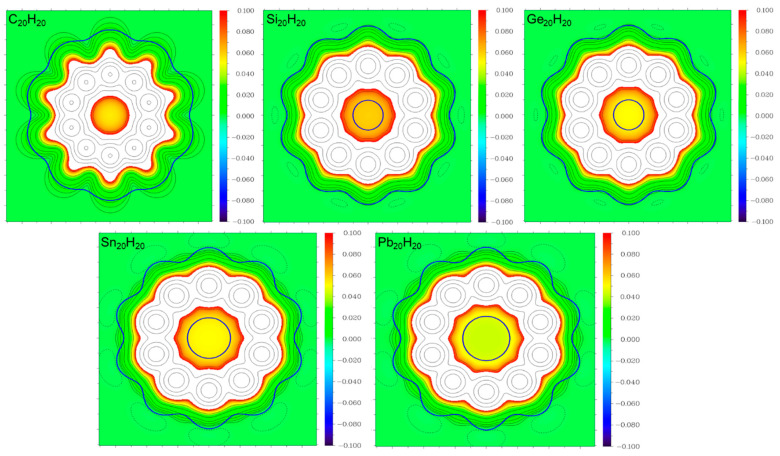
Color-filled contour line map along the XY plane of the X_20_H_20_ cages containing the center of the host molecules (blue line representing the van der Waals surface of the host).

**Figure 3:**
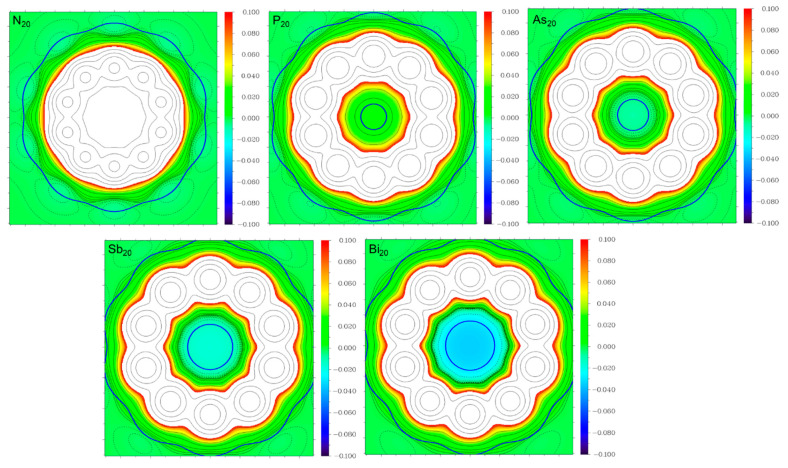
Color-filled contour line map along the XY plane of the X_20_ cages containing the center of the host molecules (blue line representing the van der Waals surface of the host).

If we compare the encapsulation energies between the elements of the same period (Figure 1), we can see that in general elements of the IV main group of the periodic table have shown lower encapsulation energies compared to the elements of the V main group. This difference is very small for the noble gases with smaller radii, but this difference increases with the increasing size of the noble gases’ radii. Our suggestion for these phenomena is that the reverse side of a XH-group in X_20_H_20_ cages has less electron density compared to the reverse side of a X_20_ cages and that larger noble gases can therefore stabilize better. That suggestion can be supported by a color-filled contour line map of charge density for the investigated hosts X_20_H_20_ and X_20_ (Figure 2 and Figure 3). Whereby, examining the cavity for the selected hosts, in a case of X_20_H_20_, there is less electron density inside the cavity in comparison to the X_20_ cages cavities.

Some optimized host–cage complexes (Ne ⊂ Si_20_H_20_, Pb_20_H_20_, Ng ⊂ Pb_20_H_20_, Kr ⊂ N_20_, Xe ⊂ N_20_, Rh ⊂ N_20_ and Ne ⊂ As_20_) show a relevant number of imaginary frequencies (Tables S2 and S3 ESI), and thus could not be trusted, while those that mention specific results are still herein reported for continuality reasons, they should be treated with a caution.

Additionally, for reason of comparison, encapsulation capabilities of two selected cages (Pb_20_H_20_ and Bi_20_) were tested on two more functionals, with the results presented in Table 3. Comparing these values with energies presented in Table 1 and Table 2 for the same systems there is an obvious difference in quantifying the encapsulation energy depending on the theory level used. While in the case of the smaller noble gases He and Ne, the difference is in the error margin (~1 kcal/mol); for the Ar noble gas this difference becomes significant, and gradually increases with the increasing of the noble gases’ radii. Also notable is that in the case of Bi_20_, the host ωB97XD functional reproduced a minimum of energies for the noble gases’ series (Ar and Kr), this minimum is absent in the case of APFD and B3LYP-GD3BJ functionals, where with the increasing of the noble gases radii we have a gradual increase of the complexation energy release upon encapsulation.

**Figure 4:**
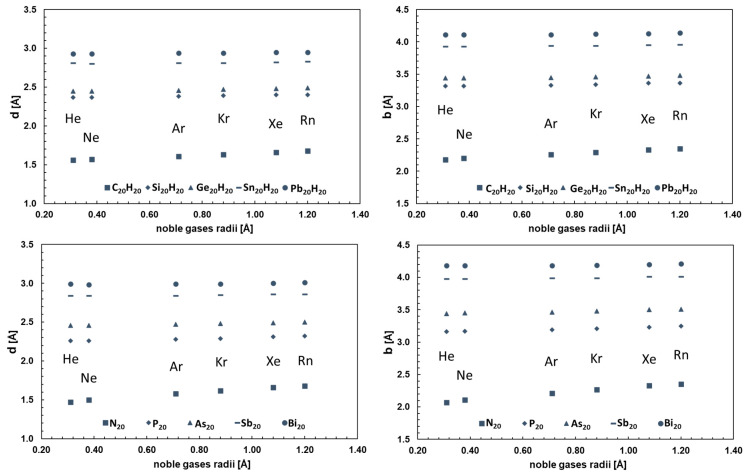
Calculated (ωB97XD/def2-SVP/SVPfit) geometric properties (*d* and *b*) in [Ng ⊂ Host] complexes plotted against the noble gas radii.

Geometric changes upon hosting a guest species can reveal insight into the flexibility of a host and its adaptability to accommodate a noble gas. In the case of the here selected dodecahedrane cages due to their symmetry, we selected two relevant structural parameters to describe their conformational change: bond distance between the atoms that are making a host (*d*; distance between the X adjacent atoms of a representative host) and the distance between one atom of the host to the hosted noble gas (*b*—distance between the X atom of the host and the hosted Ng atom). These geometric properties for the investigated hosts X_20_H_20_ and X_20_ are summarized in Table 4 and Table 5, and are plotted against the noble gas radii in Figure 4, respectively.

The results summarized in Table 4 and Table 5 and displayed in Figure 4 show very high rigidity for all investigated dodecahedrane hosts upon encapsulating noble gases. Basically, there is no adjustment of the host to the size (radii) of a guest noble gas, even if the noble gas with its size is right for a certain cavity of the dodecahedrane cage or not, which on the other hand is reflected in very large complexation energies (as, for example, in the case of C_20_H_20_ and N_20_, where we have E_com_ up to 709.69 kcal/mol or 989.41 kcal/mol for the Rn).

**Figure 5:**
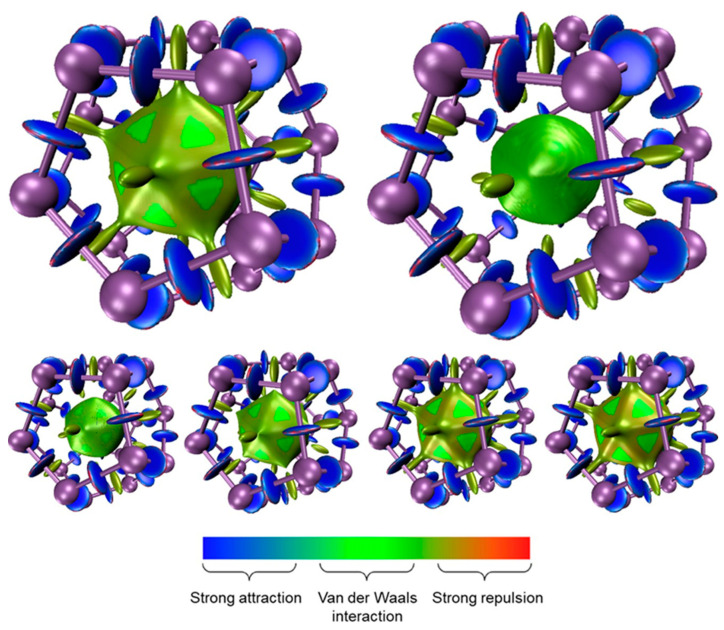
Structure of Bi_20_ with displayed non-covalent interactions according to the color bar (isoval = 0.35). **Top** row: Kr and He. **Bottom** row: Ne, Ar, Xe and Rn.

A common practice for examining non-covalent interactions (NCIs) is based on the electron density (*ρ*), the reduced gradient of the density, and the Laplacian of the density (∇^2^*ρ*) [28]. This approach enables the identification of the interactions in real space, and thus the graphical visualization of regions in which non-covalent interactions occur [40,41]. The regions of dispersion–interaction in which non-covalent interactions occur are displayed in Figure 5 and Figures S1–S9 (Supplementary Materials).

The large spatial interaction zones are in agreement with the complexation energies shown in Table 1 and Table 2, according to which the non-covalent interactions control the stabilization of the host–guest complex. The main stabilization energies are predominantly van der Waals by origin, while there is a strong repulsion in cases where the noble gases are too large for a cavity of the selected dodecahedrane cage.

## 4. Conclusions

Based on encapsulation energies gained by applying a constructed model reaction, by monitoring geometrical changes and investigating the non-covalent interactions between the studied hosts and noble gases, the size of the host plays a decisive role in the selective encapsulation of the noble gas guests. All the selected cages have proven to be very rigid with no room for adjustability upon hosting a guest species, which is reflected in energy extremes depending on the noble gas radii. From dodecahedrane cages based on the IV group of the periodic table, the Sn_20_H_20_ cage can best accommodate noble gases with a medium size radius (Ar and Kr), while the Pb_20_H_20_ dodecahedrane host is best suited for noble gases with larger radii (Xe and Rn). On the other hand, from the elements of the V main group of the periodic table, the Bi_20_ cage has shown the best results to selectively encapsulate Ar and Kr with the amount of energy released, −5.24 kcal/mol and −6.13 kcal/mol, respectively.

## Figures and Tables

**Table 1:** Calculated complexation energies (E_com_) and the BSSE energies for the X_20_H_20_ hosts based on reaction (1).

		Host/E_com_ (BSSE) *^a^* [kcal/mol]				
Noble Gas	R [Å]	C	Si	Ge	Sn	Pb
He	0.31	36.94 (0.54)	0.72 (0.37)	−0.01 (0.53)	−1.09 (0.29)	−1.31 (0.27)
Ne	0.38	103.50 (1.33)	3.31 (0.93)	−0.29 (1.24)	−3.86 (0.72)	−4.34 (0.65)
Ar	0.71	311.71 (1.59)	14.86 (1.07)	7.64 (1.59)	−5.63 (0.66)	−7.69 (0.59)
Kr	0.88	437.35 (1.71)	24.34 (1.14)	14.94 (1.83)	−6.41 (0.63)	−9.68 (0.51)
Xe	1.08	623.12 (0.58)	42.88 (0.62)	32.43 (1.45)	−2.20 (0.27)	−8.65 (0.18)
Rn	1.20	709.69 (0.70)	49.80 (0.57)	39.28 (1.49)	−2.34 (0.26)	−10.55 (0.17)

*^a^* E_com_: ωB97XD/def2-tzvp//ωB97XD/def2-svp/svpfit + ZPE(ωB97XD/def2-svp/svpfit).

**Table 2:** Calculated complexation energies (E_com_) and the BSSE energies for the X_20_ hosts based on reaction (1).

		Host/E_com_ (BSSE) *^a^* [kcal/mol]				
Noble Gas	R [Å]	N	P	As	Sb	Bi
He	0.31	57.18 (1.47)	5.62 (0.39)	0.66 (0.41)	−0.57 (0.27)	−0.73 (0.26)
Ne	0.38	165.05 (2.47)	12.70 (0.94)	3.34 (1.32)	−1.64 (0.66)	−2.78 (0.63)
Ar	0.71	474.46 (2.55)	46.16 (1.25)	17.13 (2.14)	−0.76 (0.74)	−5.24 (0.69)
Kr	0.88	659.58 (2.77)	72.46 (1.47)	30.57 (2.86)	1.36 (0.83)	−6.13 (0.69)
Xe	1.08	907.21 (1.80)	120.56 (1.06)	59.43 (2.78)	10.22 (0.71)	−2.50 (0.60)
Rn	1.20	989.41 (1.62)	143.90 (1.05)	73.03 (2.81)	12.90 (0.70)	−2.72 (0.61)

*^a^* E_com_: ωB97XD/def2-tzvp//ωB97XD/def2-svp/svpfit + ZPE(ωB97XD/def2-svp/svpfit).

**Table 3:** Calculated complexation energies (E_com_) for the Pb_20_H_20_ and Bi_20_ hosts based on reaction (1) on different theory levels.

		Host/E_com_ [kcal/mol]			
Noble Gas	R [Å]	Pb_20_H_20_ *^a^*	Bi_20_ *^a^*	Pb_20_H_20_ *^b^*	Bi_20_ *^b^*
He	0.31	−1.62	−1.25	−1.09	−0.78
Ne	0.38	−4.51	−3.51	−3.66	−3.06
Ar	0.71	−12.34	−10.28	−10.77	−9.01
Kr	0.88	−16.14	−13.45	−13.65	−11.08
Xe	1.08	−20.32	−16.46	−16.42	−11.86
Rn	1.20	−21.52	−17.06	−16.93	−11.35

*^a^* E_com_: APFD/def2-tzvp//APFD/def2-svp/svpfit + ZPE(APFD/def2-svp/svpfit); *^b^* E_com_: B3LYP-GD3BJ/def2-tzvp//B3LYP-GD3BJ/def2-svp/svpfit + ZPE(B3LYP-GD3BJ/def2-svp/svpfit).

**Table 4:** Geometric properties extracted from optimized structures at ωB97XD/def2-SVP/SVPfit theory level for X_20_H_20_ dodecahedrane cages.

		X_20_H_20_ Cage									
Noble Gas		C		Si		Ge		Sn		Pb	
	R [Å]	*d* [Å]	*b* [Å]	*d* [Å]	*b* [Å]	*d* [Å]	*b* [Å]	*d* [Å]	*b* [Å]	*d* [Å]	*b* [Å]
He	0.31	1.56	2.18	2.37	3.32	2.45	3.44	2.81	3.93	2.93	4.11
Ne	0.38	1.57	2.20	2.37	3.32	2.45	3.44	2.80	3.93	2.93	4.11
Ar	0.71	1.61	2.26	2.38	3.33	2.46	3.45	2.81	3.94	2.94	4.11
Kr	0.88	1.63	2.29	2.39	3.34	2.47	3.46	2.81	3.94	2.94	4.12
Xe	1.08	1.66	2.33	2.40	3.36	2.48	3.47	2.82	3.95	2.95	4.13
Rn	1.20	1.68	2.35	2.40	3.36	2.49	3.48	2.83	3.96	2.95	4.14
Empty host		1.55		2.37		2.45		2.81		2.93	

**Table 5:** Geometric properties extracted from optimized structures at ωB97XD/def2-SVP/SVPfit theory level for X_20_ dodecahedrane cages.

		X_20_ Cage									
Noble Gas		N		P		As		Sb		Bi	
	R [Å]	*d* [Å]	*b* [Å]	*d* [Å]	*b* [Å]	*d* [Å]	*b* [Å]	*d* [Å]	*b* [Å]	*d* [Å]	*b* [Å]
He	0.31	1.47	2.07	2.26	3.16	2.46	3.44	2.84	3.98	2.99	4.18
Ne	0.38	1.50	2.11	2.26	3.17	2.46	3.45	2.84	3.98	2.99	4.18
Ar	0.71	1.58	2.21	2.28	3.19	2.47	3.46	2.84	3.99	2.98	4.18
Kr	0.88	1.62	2.27	2.29	3.21	2.48	3.48	2.85	3.99	2.99	4.19
Xe	1.08	1.66	2.33	2.31	3.23	2.49	3.50	2.86	4.01	2.99	4.20
Rn	1.20	1.68	2.35	2.32	3.25	2.50	3.51	2.86	4.01	3.00	4.21
Empty host		1.47		2.25		2.46		2.84		2.99	

## Data Availability

The data relevant for this study are provided in the SI or can be obtained from the authors upon request.

## Supplementary Materials

The following supporting information can be downloaded at: https://www.mdpi.com/article/10.3390/molecules28155676/s1, Figure S1. Structure of C_20_H_20_ with displayed non-covalent interactions according to the color bar (isoval = 0.18). Top row: He and Rn. Bottom row: Ne, Ar, Kr and Xe; Figure S2. Structure of Si20H20 with displayed non-covalent interactions according to the color bar (isoval = 0.22). Top row: He and Rn. Bottom row: Ne, Ar, Kr and Xe; Figure S3. Structure of Ge_20_H_20_ with displayed non-covalent interactions according to the color bar (isoval = 0.3). Top row: Ne and Rn. Bottom row: He, Ar, Kr and Xe; Figure S4. Structure of Sn_20_H_20_ with displayed non-covalent interactions according to the color bar (isoval = 0.35). Top row: Kr and He. Bottom row: Ne, Ar, Xe and Rn; Figure S5. Structure of Pb_20_H_20_ with displayed non-covalent interactions according to the color bar (isoval = 0.35). Top row: Rn and He. Bottom row: Ne, Ar, Kr and Xe; Figure S6. Structure of N_20_ with displayed non-covalent interactions according to the color bar (isoval = 0.25). Top row: He and Rn. Bottom row: Ne, Ar, Kr and Xe; Figure S7. Structure of P_20_ with displayed non-covalent interactions according to the color bar (isoval = 0.3). Top row: He and Rn. Bottom row: Ne, Ar, Kr and Xe; Figure S8. Structure of As_20_ with displayed non-covalent interactions according to the color bar (isoval = 0.35). Top row: He and Rn. Bottom row: Ne, Ar, Kr and Xe; Figure S9. Structure of Sb_20_ with displayed non-covalent interactions according to the color bar (isoval = 0.37). Top row: Ne and Rn. Bottom row: He, Ar, Kr and Xe; Table S1. Optimized (ωB97XD/def2-svp/svpfit) XYZ coordinates of the structures reported in the manuscript; Table S2. Number of imaginary frequencies calculated at the ωB97XD/def2-svp/svpfit theory level for X_20_H_20_ host; Table S3. Number of imaginary frequencies calculated at the ωB97XD/def2-svp/svpfit theory level for X_20_ host; Table S4. Number of imaginary frequencies calculated on different theory levels for the Pb_20_H_20_ and Bi_20_ hosts; Table S5. Gaussian keywords input templates for the performed calculations.
