# Supplementary material for: Selective Noble Gas Inclusion in Pentagon-Dodecahedral X20-Cages
Source: Molecules. 2023 Jul 27;28(15):5676. doi: 10.3390/molecules28155676 (PMC10420277; doi:10.3390/molecules28155676)
Supplement: Supplementary file 1 [file molecules-28-05676-s001.zip › molecules-2445284-supplementary.pdf]

Supporting Information

Selective Noble Gas Inclusion in Pentagon-Dodecahedral X_{20}-Cages

Christopher Weinert [1], Dušan Ćoćić [2], Ralph Puchta [1,3,4,5,*] and Rudi van Eldik [3,6,*]

[1] Fakultät Angewandte Mathematik, Physik und Allgemeinwissenschaften, Technische Hochschule Nuremberg Georg Simon Ohm, Keßlerplatz 12, 90489 Nuremberg, Germany
[2] Department of Chemistry, Faculty of Science, University of Kragujevac, Radoja Domanovića 12, P.O. Box 60, 34000 Kragujevac, Serbia
[3] Inorganic Chemistry, Department of Chemistry and Pharmacy, University of Erlangen-Nuremberg, Egerlandstr. 1, 91058 Erlangen, Germany
[4] Central Institute for Scientific Computing (CISC), University of Erlangen-Nuremberg, Martensstr. 5a, 91058 Erlangen, Germany
[5] Computer Chemistry Center, Department of Chemistry and Pharmacy, University of Erlangen-Nuremberg, Nägelsbachstr. 25, 91052 Erlangen, Germany
[6] Faculty of Chemistry, Nicolaus Copernicus University in Toruń, Gagarina 7, 87-100 Toruń, Poland
* Correspondence: ralph.puchta@fau.de (R.P.); rudi.vaneldik@fau.de (R.v.E.)
† Dedicated to Prof. Dr. Thomas Lauterbach on the Occasion of his 60th birthday.

Table S1. Optimized (ωB97XD/ def2-svp/svpfit) XYZ coordinates of the structures reported in the manuscript.

$C_{20}H_{20}$

C -1.252105 -0.406833 1.723374
C 1.252105 1.723374 0.406833
C 1.252105 -1.723374 -0.406833
C 1.252105 -0.406833 1.723374
C 0.000000 -2.130208 0.406833
C -0.773843 1.065104 1.723374
C 2.025948 0.658270 -0.406833
C 0.000000 2.130208 -0.406833
C 0.773843 -1.065104 -1.723374
C -2.025948 -0.658270 0.406833
C 1.252105 0.406833 -1.723374
C -1.252105 -1.723374 -0.406833
C -1.252105 1.723374 0.406833
C -1.252105 0.406833 -1.723374
C -0.773843 -1.065104 -1.723374
C -2.025948 0.658270 -0.406833
C -0.000000 1.316541 -1.723374
C 2.025948 -0.658270 0.406833
C -0.000000 -1.316541 1.723374
C 0.773843 1.065104 1.723374
H 0.000000 3.209823 -0.613022
H 1.166036 -1.604911 -2.596801
H -3.052723 -0.991890 0.613022
H 1.886687 0.613022 -2.596801
H -1.886687 -2.596801 -0.613022
H -1.886687 2.596801 0.613022
H -1.886687 0.613022 -2.596801

H -1.166036 -1.604911 -2.596801
H -3.052723 0.991890 -0.613022
H -0.000000 1.983780 -2.596801
H 3.052723 0.991890 -0.613022
H 0.000000 -3.209823 0.613022
H -1.166036 1.604911 2.596801
H -1.886687 -0.613022 2.596801
H 1.886687 2.596801 0.613022
H 1.886687 -2.596801 -0.613022
H 1.886687 -0.613022 2.596801
H -0.000000 -1.983780 2.596801
H 1.166036 1.604911 2.596801
H 3.052723 -0.991890 0.613022

$C_{20}H_{20} \subset He$
He 0.000000 0.000000 0.000000
C -1.258861 -0.409029 1.732673
C 1.258861 1.732673 0.409029
C 1.258861 -1.732673 -0.409029
C 1.258861 -0.409029 1.732673
C -0.000000 -2.141701 0.409029
C -0.778019 1.070851 1.732673
C 2.036879 0.661822 -0.409029
C -0.000000 2.141701 -0.409029
C 0.778019 -1.070851 -1.732673
C -2.036879 -0.661822 0.409029
C 1.258861 0.409029 -1.732673
C -1.258861 -1.732673 -0.409029
C -1.258861 1.732673 0.409029
C -1.258861 0.409029 -1.732673

C -0.778019 -1.070851 -1.732673
C -2.036879 0.661822 -0.409029
C -0.000000 1.323644 -1.732673
C 2.036879 -0.661822 0.409029
C -0.000000 -1.323644 1.732673
C 0.778019 1.070851 1.732673
H -0.000000 3.220980 -0.615152
H 1.170089 -1.610490 -2.605827
H -3.063334 -0.995338 0.615152
H 1.893244 0.615152 -2.605827
H -1.893244 -2.605827 -0.615152
H -1.893244 2.605827 0.615152
H -1.893244 0.615152 -2.605827
H -1.170089 -1.610490 -2.605827
H -3.063334 0.995338 -0.615152
H -0.000000 1.990675 -2.605827
H 3.063334 0.995338 -0.615152
H -0.000000 -3.220980 0.615152
H -1.170089 1.610490 2.605827
H -1.893244 -0.615152 2.605827
H 1.893244 2.605827 0.615152
H 1.893244 -2.605827 -0.615152
H 1.893244 -0.615152 2.605827
H -0.000000 -1.990675 2.605827
H 1.170089 1.610490 2.605827
H 3.063334 -0.995338 0.615152

$C_{20}H_{20} \subset Ne$
Ne 0.000000 0.000000 0.000000
C -1.271734 -0.413211 1.750392

C 1.271734 1.750392 0.413211
C 1.271734 -1.750392 -0.413211
C 1.271734 -0.413211 1.750392
C 0.000000 -2.163603 0.413211
C -0.785975 1.081801 1.750392
C 2.057709 0.668590 -0.413211
C 0.000000 2.163603 -0.413211
C 0.785975 -1.081801 -1.750392
C -2.057709 -0.668590 0.413211
C 1.271734 0.413211 -1.750392
C -1.271734 -1.750392 -0.413211
C -1.271734 1.750392 0.413211
C -1.271734 0.413211 -1.750392
C -0.785975 -1.081801 -1.750392
C -2.057709 0.668590 -0.413211
C 0.000000 1.337180 -1.750392
C 2.057709 -0.668590 0.413211
C -0.000000 -1.337180 1.750392
C 0.785975 1.081801 1.750392
H 0.000000 3.242417 -0.619246
H 1.177877 -1.621208 -2.623170
H -3.083721 -1.001962 0.619246
H 1.905845 0.619246 -2.623170
H -1.905845 -2.623170 -0.619246
H -1.905845 2.623170 0.619246
H -1.905845 0.619246 -2.623170
H -1.177877 -1.621208 -2.623170
H -3.083721 1.001962 -0.619246
H 0.000000 2.003924 -2.623170
H 3.083721 1.001962 -0.619246
H 0.000000 -3.242417 0.619246

H -1.177877 1.621208 2.623170
H -1.905845 -0.619246 2.623170
H 1.905845 2.623170 0.619246
H 1.905845 -2.623170 -0.619246
H 1.905845 -0.619246 2.623170
H -0.000000 -2.003924 2.623170
H 1.177877 1.621208 2.623170
H 3.083721 -1.001962 0.619246

$C_{20}H_{20} \subset Ar$
Ar 0.000000 0.000000 0.000000
C -1.304012 -0.423699 1.794819
C 1.304012 1.794819 0.423699
C 1.304012 -1.794819 -0.423699
C 1.304012 -0.423699 1.794819
C -0.000000 -2.218518 0.423699
C -0.805924 1.109259 1.794819
C 2.109936 0.685560 -0.423699
C 0.000000 2.218518 -0.423699
C 0.805924 -1.109259 -1.794819
C -2.109936 -0.685560 0.423699
C 1.304012 0.423699 -1.794819
C -1.304012 -1.794819 -0.423699
C -1.304012 1.794819 0.423699
C -1.304012 0.423699 -1.794819
C -0.805924 -1.109259 -1.794819
C -2.109936 0.685560 -0.423699
C 0.000000 1.371119 -1.794819
C 2.109936 -0.685560 0.423699
C -0.000000 -1.371119 1.794819

C 0.805924 1.109259 1.794819
H 0.000000 3.299477 -0.630144
H 1.198605 -1.649738 -2.669333
H -3.137989 -1.019594 0.630144
H 1.939384 0.630144 -2.669333
H -1.939384 -2.669333 -0.630144
H -1.939384 2.669333 0.630144
H -1.939384 0.630144 -2.669333
H -1.198605 -1.649738 -2.669333
H -3.137989 1.019594 -0.630144
H 0.000000 2.039189 -2.669333
H 3.137989 1.019594 -0.630144
H -0.000000 -3.299477 0.630144
H -1.198605 1.649738 2.669333
H -1.939384 -0.630144 2.669333
H 1.939384 2.669333 0.630144
H 1.939384 -2.669333 -0.630144
H 1.939384 -0.630144 2.669333
H -0.000000 -2.039189 2.669333
H 1.198605 1.649738 2.669333
H 3.137989 -1.019594 0.630144

$C_{20}H_{20} \subset Kr$
Kr 0.000000 0.000000 0.000000
C -1.32186 -0.4294981.819384
C 1.32186 1.819384 0.429498
C 1.32186 -1.819384 -0.429498
C 1.32186 -0.429498 1.819384
C 0 -2.248882 0.429498
C -0.816954 1.124441 1.819384

C	2.138814	0.694943	-0.429498
C	0	2.248882	-0.429498
C	0.816954	-1.124441	-1.819384
C	-2.138814	-0.694943	0.429498
C	1.32186	0.429498	-1.819384
C	-1.32186	-1.819384	-0.429498
C	-1.32186	1.819384	0.429498
C	-1.32186	0.429498	-1.819384
C	-0.816954	-1.124441	-1.819384
C	-2.138814	0.694943	-0.429498
C	0	1.389886	-1.819384
C	2.138814	-0.694943	0.429498
C	0	-1.389886	1.819384
C	0.816954	1.124441	1.819384
H	0	3.332732	-0.636495
H	1.210686	-1.666366	-2.696237
H	-3.169616	-1.029871	0.636495
H	1.958931	0.636495	-2.696237
H	-1.958931	-2.696237	-0.636495
H	-1.958931	2.696237	0.636495
H	-1.958931	0.636495	-2.696237
H	-1.210686	-1.666366	-2.696237
H	-3.169616	1.029871	-0.636495
H	0	2.059742	-2.696237
H	3.169616	1.029871	-0.636495
H	0	-3.332732	0.636495
H	-1.210686	1.666366	2.696237
H	-1.958931	-0.636495	2.696237
H	1.958931	2.696237	0.636495
H	1.958931	-2.696237	-0.636495
H	1.958931	-0.636495	2.696237

H 0 -2.059742 2.696237

H 1.210686 1.666366 2.696237

H 3.169616 -1.029871 0.636495

$C_{20}H_{20} \subset Xe$

Xe	0	0	0
C	-1.342418	-0.436178	1.847679
C	1.342418	1.847679	0.436178
C	1.342418	-1.847679	-0.436178
C	1.342418	-0.436178	1.847679
C	0	-2.283857	0.436178
C	-0.82966	1.141929	1.847679
C	2.172077	0.705751	-0.436178
C	0	2.283857	-0.436178
C	0.82966	-1.141929	-1.847679
C	-2.172077	-0.705751	0.436178
C	1.342418	0.436178	-1.847679
C	-1.342418	-1.847679	-0.436178
C	-1.342418	1.847679	0.436178
C	-1.342418	0.436178	-1.847679
C	-0.82966	-1.141929	-1.847679
C	-2.172077	0.705751	-0.436178
C	0	1.411501	-1.847679
C	2.172077	-0.705751	0.436178
C	0	-1.411501	1.847679
C	0.82966	1.141929	1.847679
H	0	3.372623	-0.644114
H	1.225177	-1.686312	-2.728509
H	-3.207555	-1.042198	0.644114
H	1.982378	0.644114	-2.728509

H	-1.982378	-2.728509	-0.644114
H	-1.982378	2.728509	0.644114
H	-1.982378	0.644114	-2.728509
H	-1.225177	-1.686312	-2.728509
H	-3.207555	1.042198	-0.644114
H	0	2.084396	-2.728509
H	3.207555	1.042198	-0.644114
H	0	-3.372623	0.644114
H	-1.225177	1.686312	2.728509
H	-1.982378	-0.644114	2.728509
H	1.982378	2.728509	0.644114
H	1.982378	-2.728509	-0.644114
H	1.982378	-0.644114	2.728509
H	0	-2.084396	2.728509
H	1.225177	1.686312	2.728509
H	3.207555	-1.042198	0.644114

$C_{20}H_{20} \subset Rn$

Rn	0	0	0
C	-1.355594	-0.440459	1.865815
C	1.355594	1.865815	0.440459
C	1.355594	-1.865815	-0.440459
C	1.355594	-0.440459	1.865815
C	0	-2.306274	0.440459
C	-0.837803	1.153137	1.865815
C	2.193397	0.712678	-0.440459
C	0	2.306274	-0.440459
C	0.837803	-1.153137	-1.865815
C	-2.193397	-0.712678	0.440459
C	1.355594	0.440459	-1.865815

C	-1.355594	-1.865815	-0.440459
C	-1.355594	1.865815	0.440459
C	-1.355594	0.440459	-1.865815
C	-0.837803	-1.153137	-1.865815
C	-2.193397	0.712678	-0.440459
C	0	1.425356	-1.865815
C	2.193397	-0.712678	0.440459
C	0	-1.425356	1.865815
C	0.837803	1.153137	1.865815
H	0	3.397076	-0.648784
H	1.23406	-1.698538	-2.748292
H	-3.230811	-1.049754	0.648784
H	1.996751	0.648784	-2.748292
H	-1.996751	-2.748292	-0.648784
H	-1.996751	2.748292	0.648784
H	-1.996751	0.648784	-2.748292
H	-1.23406	-1.698538	-2.748292
H	-3.230811	1.049754	-0.648784
H	0	2.099508	-2.748292
H	3.230811	1.049754	-0.648784
H	0	-3.397076	0.648784
H	-1.23406	1.698538	2.748292
H	-1.996751	-0.648784	2.748292
H	1.996751	2.748292	0.648784
H	1.996751	-2.748292	-0.648784
H	1.996751	-0.648784	2.748292
H	0	-2.099508	2.748292
H	1.23406	1.698538	2.748292
H	3.230811	-1.049754	0.648784

$Si_{20}H_{20}$

Si	-1.914952	-0.622206	2.635706
Si	1.914952	2.635706	0.622206
Si	1.914952	-2.635706	-0.622206
Si	1.914952	-0.622206	2.635706
Si	0	-3.257912	0.622206
Si	-1.183506	1.628956	2.635706
Si	3.098458	1.00675	-0.622206
Si	0	3.257912	-0.622206
Si	1.183506	-1.628956	-2.635706
Si	-3.098458	-1.00675	0.622206
Si	1.914952	0.622206	-2.635706
Si	-1.914952	-2.635706	-0.622206
Si	-1.914952	2.635706	0.622206
Si	-1.914952	0.622206	-2.635706
Si	-1.183506	-1.628956	-2.635706
Si	-3.098458	1.00675	-0.622206
Si	0	2.0135	-2.635706
Si	3.098458	-1.00675	0.622206
Si	0	-2.0135	2.635706
Si	1.183506	1.628956	2.635706
H	0	4.726497	-0.902681
H	1.717	-2.363248	-3.823816
H	-4.495165	-1.460568	0.902681
H	2.778165	0.902681	-3.823816
H	-2.778165	-3.823816	-0.902681
H	-2.778165	3.823816	0.902681
H	-2.778165	0.902681	-3.823816
H	-1.717	-2.363248	-3.823816
H	-4.495165	1.460568	-0.902681
H	0	2.921136	-3.823816
H	4.495165	1.460568	-0.902681

H	0	-4.726497	0.902681
H	-1.717	2.363248	3.823816
H	-2.778165	-0.902681	3.823816
H	2.778165	3.823816	0.902681
H	2.778165	-3.823816	-0.902681
H	2.778165	-0.902681	3.823816
H	0	-2.921136	3.823816
H	1.717	2.363248	3.823816
H	4.495165	-1.460568	0.902681

$Si_{20}H_{20} \subset He$

He	0	0	0
Si	-1.915933	-0.622525	2.637056
Si	1.915933	2.637056	0.622525
Si	1.915933	-2.637056	-0.622525
Si	1.915933	-0.622525	2.637056
Si	0	-3.259581	0.622525
Si	-1.184112	1.62979	2.637056
Si	3.100045	1.007266	-0.622525
Si	0	3.259581	-0.622525
Si	1.184112	-1.62979	-2.637056
Si	-3.100045	-1.007266	0.622525
Si	1.915933	0.622525	-2.637056
Si	-1.915933	-2.637056	-0.622525
Si	-1.915933	2.637056	0.622525
Si	-1.915933	0.622525	-2.637056
Si	-1.184112	-1.62979	-2.637056
Si	-3.100045	1.007266	-0.622525
Si	0	2.014532	-2.637056
Si	3.100045	-1.007266	0.622525

Si	0	-2.014532	2.637056
Si	1.184112	1.62979	2.637056
H	0	4.727809	-0.902931
H	1.717477	-2.363905	-3.824878
H	-4.496414	-1.460973	0.902931
H	2.778936	0.902931	-3.824878
H	-2.778936	-3.824878	-0.902931
H	-2.778936	3.824878	0.902931
H	-2.778936	0.902931	-3.824878
H	-1.717477	-2.363905	-3.824878
H	-4.496414	1.460973	-0.902931
H	0	2.921947	-3.824878
H	4.496414	1.460973	-0.902931
H	0	-4.727809	0.902931
H	-1.717477	2.363905	3.824878
H	-2.778936	-0.902931	3.824878
H	2.778936	3.824878	0.902931
H	2.778936	-3.824878	-0.902931
H	2.778936	-0.902931	3.824878
H	0	-2.921947	3.824878
H	1.717477	2.363905	3.824878
H	4.496414	-1.460973	0.902931

$Si_{20}H_{20} \subset Ne$

Ne	0	0	0
Si	-1.917032	-0.622881	2.638568
Si	1.917032	2.638568	0.622881
Si	1.917032	-2.638568	-0.622881
Si	1.917032	-0.622881	2.638568
Si	0	-3.261449	0.622881

Si	-1.184791	1.630725	2.638568
Si	3.101823	1.007843	-0.622881
Si	0	3.261449	-0.622881
Si	1.184791	-1.630725	-2.638568
Si	-3.101823	-1.007843	0.622881
Si	1.917032	0.622881	-2.638568
Si	-1.917032	-2.638568	-0.622881
Si	-1.917032	2.638568	0.622881
Si	-1.917032	0.622881	-2.638568
Si	-1.184791	-1.630725	-2.638568
Si	-3.101823	1.007843	-0.622881
Si	0	2.015687	-2.638568
Si	3.101823	-1.007843	0.622881
Si	0	-2.015687	2.638568
Si	1.184791	1.630725	2.638568
H	0	4.729535	-0.903261
H	1.718104	-2.364768	-3.826275
H	-4.498056	-1.461507	0.903261
H	2.779951	0.903261	-3.826275
H	-2.779951	-3.826275	-0.903261
H	-2.779951	3.826275	0.903261
H	-2.779951	0.903261	-3.826275
H	-1.718104	-2.364768	-3.826275
H	-4.498056	1.461507	-0.903261
H	0	2.923014	-3.826275
H	4.498056	1.461507	-0.903261
H	0	-4.729535	0.903261
H	-1.718104	2.364768	3.826275
H	-2.779951	-0.903261	3.826275
H	2.779951	3.826275	0.903261
H	2.779951	-3.826275	-0.903261

H	2.779951	-0.903261	3.826275
H	0	-2.923014	3.826275
H	1.718104	2.364768	3.826275
H	4.498056	-1.461507	0.903261

$Si_{20}H_{20} \subset Ar$

Ar	0	0	0
Si	-1.923931	-0.625123	2.648064
Si	1.923931	2.648064	0.625123
Si	1.923931	-2.648064	-0.625123
Si	1.923931	-0.625123	2.648064
Si	0	-3.273187	0.625123
Si	-1.189055	1.636593	2.648064
Si	3.112986	1.01147	-0.625123
Si	0	3.273187	-0.625123
Si	1.189055	-1.636593	-2.648064
Si	-3.112986	-1.01147	0.625123
Si	1.923931	0.625123	-2.648064
Si	-1.923931	-2.648064	-0.625123
Si	-1.923931	2.648064	0.625123
Si	-1.923931	0.625123	-2.648064
Si	-1.189055	-1.636593	-2.648064
Si	-3.112986	1.01147	-0.625123
Si	0	2.022941	-2.648064
Si	3.112986	-1.01147	0.625123
Si	0	-2.022941	2.648064
Si	1.189055	1.636593	2.648064
H	0	4.740989	-0.905448
H	1.722265	-2.370495	-3.835541
H	-4.508949	-1.465046	0.905448

H	2.786683	0.905448	-3.835541
H	-2.786683	-3.835541	-0.905448
H	-2.786683	3.835541	0.905448
H	-2.786683	0.905448	-3.835541
H	-1.722265	-2.370495	-3.835541
H	-4.508949	1.465046	-0.905448
H	0	2.930092	-3.835541
H	4.508949	1.465046	-0.905448
H	0	-4.740989	0.905448
H	-1.722265	2.370495	3.835541
H	-2.786683	-0.905448	3.835541
H	2.786683	3.835541	0.905448
H	2.786683	-3.835541	-0.905448
H	2.786683	-0.905448	3.835541
H	0	-2.930092	3.835541
H	1.722265	2.370495	3.835541
H	4.508949	-1.465046	0.905448

$Si_{20}H_{20} \subset Kr$

Kr	0	0	0
Si	-1.931114	-0.627457	2.657951
Si	1.931114	2.657951	0.627457
Si	1.931114	-2.657951	-0.627457
Si	1.931114	-0.627457	2.657951
Si	0	-3.285408	0.627457
Si	-1.193494	1.642704	2.657951
Si	3.124608	1.015247	-0.627457
Si	0	3.285408	-0.627457
Si	1.193494	-1.642704	-2.657951
Si	-3.124608	-1.015247	0.627457

Si	1.931114	0.627457	-2.657951
Si	-1.931114	-2.657951	-0.627457
Si	-1.931114	2.657951	0.627457
Si	-1.931114	0.627457	-2.657951
Si	-1.193494	-1.642704	-2.657951
Si	-3.124608	1.015247	-0.627457
Si	0	2.030494	-2.657951
Si	3.124608	-1.015247	0.627457
Si	0	-2.030494	2.657951
Si	1.193494	1.642704	2.657951
H	0	4.753216	-0.907783
H	1.726707	-2.376608	-3.845432
H	-4.520577	-1.468824	0.907783
H	2.79387	0.907783	-3.845432
H	-2.79387	-3.845432	-0.907783
H	-2.79387	3.845432	0.907783
H	-2.79387	0.907783	-3.845432
H	-1.726707	-2.376608	-3.845432
H	-4.520577	1.468824	-0.907783
H	0	2.937649	-3.845432
H	4.520577	1.468824	-0.907783
H	0	-4.753216	0.907783
H	-1.726707	2.376608	3.845432
H	-2.79387	-0.907783	3.845432
H	2.79387	3.845432	0.907783
H	2.79387	-3.845432	-0.907783
H	2.79387	-0.907783	3.845432
H	0	-2.937649	3.845432
H	1.726707	2.376608	3.845432
H	4.520577	-1.468824	0.907783

$Si_{20}H_{20} \subset Xe$

Xe	0	0	0
Si	-1.939288	-0.630113	2.669201
Si	1.939288	2.669201	0.630113
Si	1.939288	-2.669201	-0.630113
Si	1.939288	-0.630113	2.669201
Si	0	-3.299314	0.630113
Si	-1.198546	1.649657	2.669201
Si	3.137834	1.019544	-0.630113
Si	0	3.299314	-0.630113
Si	1.198546	-1.649657	-2.669201
Si	-3.137834	-1.019544	0.630113
Si	1.939288	0.630113	-2.669201
Si	-1.939288	-2.669201	-0.630113
Si	-1.939288	2.669201	0.630113
Si	-1.939288	0.630113	-2.669201
Si	-1.198546	-1.649657	-2.669201
Si	-3.137834	1.019544	-0.630113
Si	0	2.039088	-2.669201
Si	3.137834	-1.019544	0.630113
Si	0	-2.039088	2.669201
Si	1.198546	1.649657	2.669201
H	0	4.767448	-0.910502
H	1.731877	-2.383724	-3.856947
H	-4.534113	-1.473222	0.910502
H	2.802236	0.910502	-3.856947
H	-2.802236	-3.856947	-0.910502
H	-2.802236	3.856947	0.910502
H	-2.802236	0.910502	-3.856947
H	-1.731877	-2.383724	-3.856947

H	-4.534113	1.473222	-0.910502
H	0	2.946445	-3.856947
H	4.534113	1.473222	-0.910502
H	0	-4.767448	0.910502
H	-1.731877	2.383724	3.856947
H	-2.802236	-0.910502	3.856947
H	2.802236	3.856947	0.910502
H	2.802236	-3.856947	-0.910502
H	2.802236	-0.910502	3.856947
H	0	-2.946445	3.856947
H	1.731877	2.383724	3.856947
H	4.534113	-1.473222	0.910502

$Si_{20}H_{20} \subset Rn$

Rn	0	0	0
Si	-1.942698	-0.631221	2.673894
Si	1.942698	2.673894	0.631221
Si	1.942698	-2.673894	-0.631221
Si	1.942698	-0.631221	2.673894
Si	0	-3.305115	0.631221
Si	-1.200653	1.652558	2.673894
Si	3.143351	1.021337	-0.631221
Si	0	3.305115	-0.631221
Si	1.200653	-1.652558	-2.673894
Si	-3.143351	-1.021337	0.631221
Si	1.942698	0.631221	-2.673894
Si	-1.942698	-2.673894	-0.631221
Si	-1.942698	2.673894	0.631221
Si	-1.942698	0.631221	-2.673894
Si	-1.200653	-1.652558	-2.673894

Si	-3.143351	1.021337	-0.631221
Si	0	2.042673	-2.673894
Si	3.143351	-1.021337	0.631221
Si	0	-2.042673	2.673894
Si	1.200653	1.652558	2.673894
H	0	4.773569	-0.911671
H	1.7341	-2.386784	-3.861898
H	-4.539934	-1.475114	0.911671
H	2.805833	0.911671	-3.861898
H	-2.805833	-3.861898	-0.911671
H	-2.805833	3.861898	0.911671
H	-2.805833	0.911671	-3.861898
H	-1.7341	-2.386784	-3.861898
H	-4.539934	1.475114	-0.911671
H	0	2.950228	-3.861898
H	4.539934	1.475114	-0.911671
H	0	-4.773569	0.911671
H	-1.7341	2.386784	3.861898
H	-2.805833	-0.911671	3.861898
H	2.805833	3.861898	0.911671
H	2.805833	-3.861898	-0.911671
H	2.805833	-0.911671	3.861898
H	0	-2.950228	3.861898
H	1.7341	2.386784	3.861898
H	4.539934	-1.475114	0.911671

$Ge_{20}H_{20}$

Ge	-1.982484	-0.644148	2.728655
Ge	1.982484	2.728655	0.644148
Ge	1.982484	-2.728655	-0.644148
Ge	1.982484	-0.644148	2.728655

Ge	0	-3.372803	0.644148
Ge	-1.225242	1.686401	2.728655
Ge	3.207726	1.042253	-0.644148
Ge	0	3.372803	-0.644148
Ge	1.225242	-1.686401	-2.728655
Ge	-3.207726	-1.042253	0.644148
Ge	1.982484	0.644148	-2.728655
Ge	-1.982484	-2.728655	-0.644148
Ge	-1.982484	2.728655	0.644148
Ge	-1.982484	0.644148	-2.728655
Ge	-1.225242	-1.686401	-2.728655
Ge	-3.207726	1.042253	-0.644148
Ge	0	2.084507	-2.728655
Ge	3.207726	-1.042253	0.644148
Ge	0	-2.084507	2.728655
Ge	1.225242	1.686401	2.728655
H	0	4.887037	-0.933341
H	1.77532	-2.443518	-3.953696
H	-4.647848	-1.510177	0.933341
H	2.872528	0.933341	-3.953696
H	-2.872528	-3.953696	-0.933341
H	-2.872528	3.953696	0.933341
H	-2.872528	0.933341	-3.953696
H	-1.77532	-2.443518	-3.953696
H	-4.647848	1.510177	-0.933341
H	0	3.020355	-3.953696
H	4.647848	1.510177	-0.933341
H	0	-4.887037	0.933341
H	-1.77532	2.443518	3.953696
H	-2.872528	-0.933341	3.953696
H	2.872528	3.953696	0.933341

H	2.872528	-3.953696	-0.933341
H	2.872528	-0.933341	3.953696
H	0	-3.020355	3.953696
H	1.77532	2.443518	3.953696
H	4.647848	-1.510177	0.933341

$Ge_{20}H_{20} \subset He$

He	0	0	0
Ge	-1.983604	-0.644512	2.730197
Ge	1.983604	2.730197	0.644512
Ge	1.983604	-2.730197	-0.644512
Ge	1.983604	-0.644512	2.730197
Ge	0	-3.374709	0.644512
Ge	-1.225935	1.687355	2.730197
Ge	3.209539	1.042843	-0.644512
Ge	0	3.374709	-0.644512
Ge	1.225935	-1.687355	-2.730197
Ge	-3.209539	-1.042843	0.644512
Ge	1.983604	0.644512	-2.730197
Ge	-1.983604	-2.730197	-0.644512
Ge	-1.983604	2.730197	0.644512
Ge	-1.983604	0.644512	-2.730197
Ge	-1.225935	-1.687355	-2.730197
Ge	-3.209539	1.042843	-0.644512
Ge	0	2.085685	-2.730197
Ge	3.209539	-1.042843	0.644512
Ge	0	-2.085685	2.730197
Ge	1.225935	1.687355	2.730197
H	0	4.888794	-0.933677
H	1.775958	-2.444397	-3.955118

H	-4.64952	-1.51072	0.933677
H	2.873561	0.933677	-3.955118
H	-2.873561	-3.955118	-0.933677
H	-2.873561	3.955118	0.933677
H	-2.873561	0.933677	-3.955118
H	-1.775958	-2.444397	-3.955118
H	-4.64952	1.51072	-0.933677
H	0	3.021441	-3.955118
H	4.64952	1.51072	-0.933677
H	0	-4.888794	0.933677
H	-1.775958	2.444397	3.955118
H	-2.873561	-0.933677	3.955118
H	2.873561	3.955118	0.933677
H	2.873561	-3.955118	-0.933677
H	2.873561	-0.933677	3.955118
H	0	-3.021441	3.955118
H	1.775958	2.444397	3.955118
H	4.64952	-1.51072	0.933677

$Ge_{20}H_{20} \subset Ne$

Ne	0	0	0
Ge	-1.984042	-0.644654	2.7308
Ge	1.984042	2.7308	0.644654
Ge	1.984042	-2.7308	-0.644654
Ge	1.984042	-0.644654	2.7308
Ge	0	-3.375454	0.644654
Ge	-1.226206	1.687727	2.7308
Ge	3.210248	1.043073	-0.644654
Ge	0	3.375454	-0.644654
Ge	1.226206	-1.687727	-2.7308

Ge	-3.210248	-1.043073	0.644654
Ge	1.984042	0.644654	-2.7308
Ge	-1.984042	-2.7308	-0.644654
Ge	-1.984042	2.7308	0.644654
Ge	-1.984042	0.644654	-2.7308
Ge	-1.226206	-1.687727	-2.7308
Ge	-3.210248	1.043073	-0.644654
Ge	0	2.086145	-2.7308
Ge	3.210248	-1.043073	0.644654
Ge	0	-2.086145	2.7308
Ge	1.226206	1.687727	2.7308
H	0	4.889469	-0.933805
H	1.776203	-2.444734	-3.955663
H	-4.650161	-1.510929	0.933805
H	2.873957	0.933805	-3.955663
H	-2.873957	-3.955663	-0.933805
H	-2.873957	3.955663	0.933805
H	-2.873957	0.933805	-3.955663
H	-1.776203	-2.444734	-3.955663
H	-4.650161	1.510929	-0.933805
H	0	3.021858	-3.955663
H	4.650161	1.510929	-0.933805
H	0	-4.889469	0.933805
H	-1.776203	2.444734	3.955663
H	-2.873957	-0.933805	3.955663
H	2.873957	3.955663	0.933805
H	2.873957	-3.955663	-0.933805
H	2.873957	-0.933805	3.955663
H	0	-3.021858	3.955663
H	1.776203	2.444734	3.955663
H	4.650161	-1.510929	0.933805

$Ge_{20}H_{20} \subset Ar$

Ar	0	0	0
Ge	-1.992727	-0.647476	2.742754
Ge	1.992727	2.742754	0.647476
Ge	1.992727	-2.742754	-0.647476
Ge	1.992727	-0.647476	2.742754
Ge	0	-3.39023	0.647476
Ge	-1.231573	1.695115	2.742754
Ge	3.224301	1.047639	-0.647476
Ge	0	3.39023	-0.647476
Ge	1.231573	-1.695115	-2.742754
Ge	-3.224301	-1.047639	0.647476
Ge	1.992727	0.647476	-2.742754
Ge	-1.992727	-2.742754	-0.647476
Ge	-1.992727	2.742754	0.647476
Ge	-1.992727	0.647476	-2.742754
Ge	-1.231573	-1.695115	-2.742754
Ge	-3.224301	1.047639	-0.647476
Ge	0	2.095278	-2.742754
Ge	3.224301	-1.047639	0.647476
Ge	0	-2.095278	2.742754
Ge	1.231573	1.695115	2.742754
H	0	4.904431	-0.936663
H	1.781639	-2.452215	-3.967768
H	-4.664391	-1.515552	0.936663
H	2.882752	0.936663	-3.967768
H	-2.882752	-3.967768	-0.936663
H	-2.882752	3.967768	0.936663
H	-2.882752	0.936663	-3.967768

H	-1.781639	-2.452215	-3.967768
H	-4.664391	1.515552	-0.936663
H	0	3.031105	-3.967768
H	4.664391	1.515552	-0.936663
H	0	-4.904431	0.936663
H	-1.781639	2.452215	3.967768
H	-2.882752	-0.936663	3.967768
H	2.882752	3.967768	0.936663
H	2.882752	-3.967768	-0.936663
H	2.882752	-0.936663	3.967768
H	0	-3.031105	3.967768
H	1.781639	2.452215	3.967768
H	4.664391	-1.515552	0.936663

$Ge_{20}H_{20} \subset Kr$

Kr	0	0	0
Ge	-1.997349	-0.648978	2.749115
Ge	1.997349	2.749115	0.648978
Ge	1.997349	-2.749115	-0.648978
Ge	1.997349	-0.648978	2.749115
Ge	0	-3.398092	0.648978
Ge	-1.234429	1.699046	2.749115
Ge	3.231778	1.050068	-0.648978
Ge	0	3.398092	-0.648978
Ge	1.234429	-1.699046	-2.749115
Ge	-3.231778	-1.050068	0.648978
Ge	1.997349	0.648978	-2.749115
Ge	-1.997349	-2.749115	-0.648978
Ge	-1.997349	2.749115	0.648978
Ge	-1.997349	0.648978	-2.749115

Ge	-1.234429	-1.699046	-2.749115
Ge	-3.231778	1.050068	-0.648978
Ge	0	2.100137	-2.749115
Ge	3.231778	-1.050068	0.648978
Ge	0	-2.100137	2.749115
Ge	1.234429	1.699046	2.749115
H	0	4.912556	-0.938215
H	1.78459	-2.456278	-3.974341
H	-4.672118	-1.518063	0.938215
H	2.887528	0.938215	-3.974341
H	-2.887528	-3.974341	-0.938215
H	-2.887528	3.974341	0.938215
H	-2.887528	0.938215	-3.974341
H	-1.78459	-2.456278	-3.974341
H	-4.672118	1.518063	-0.938215
H	0	3.036127	-3.974341
H	4.672118	1.518063	-0.938215
H	0	-4.912556	0.938215
H	-1.78459	2.456278	3.974341
H	-2.887528	-0.938215	3.974341
H	2.887528	3.974341	0.938215
H	2.887528	-3.974341	-0.938215
H	2.887528	-0.938215	3.974341
H	0	-3.036127	3.974341
H	1.78459	2.456278	3.974341
H	4.672118	-1.518063	0.938215

$Ge_{20}H_{20} \subset Xe$

Xe	0	0	0
Ge	-2.005428	-0.651603	2.760234

Ge	2.005428	2.760234	0.651603
Ge	2.005428	-2.760234	-0.651603
Ge	2.005428	-0.651603	2.760234
Ge	0	-3.411837	0.651603
Ge	-1.239422	1.705919	2.760234
Ge	3.24485	1.054316	-0.651603
Ge	0	3.411837	-0.651603
Ge	1.239422	-1.705919	-2.760234
Ge	-3.24485	-1.054316	0.651603
Ge	2.005428	0.651603	-2.760234
Ge	-2.005428	-2.760234	-0.651603
Ge	-2.005428	2.760234	0.651603
Ge	-2.005428	0.651603	-2.760234
Ge	-1.239422	-1.705919	-2.760234
Ge	-3.24485	1.054316	-0.651603
Ge	0	2.108631	-2.760234
Ge	3.24485	-1.054316	0.651603
Ge	0	-2.108631	2.760234
Ge	1.239422	1.705919	2.760234
H	0	4.927013	-0.940976
H	1.789842	-2.463507	-3.986037
H	-4.685868	-1.522531	0.940976
H	2.896026	0.940976	-3.986037
H	-2.896026	-3.986037	-0.940976
H	-2.896026	3.986037	0.940976
H	-2.896026	0.940976	-3.986037
H	-1.789842	-2.463507	-3.986037
H	-4.685868	1.522531	-0.940976
H	0	3.045062	-3.986037
H	4.685868	1.522531	-0.940976
H	0	-4.927013	0.940976

H	-1.789842	2.463507	3.986037
H	-2.896026	-0.940976	3.986037
H	2.896026	3.986037	0.940976
H	2.896026	-3.986037	-0.940976
H	2.896026	-0.940976	3.986037
H	0	-3.045062	3.986037
H	1.789842	2.463507	3.986037
H	4.685868	-1.522531	0.940976

$Ge_{20}H_{20} \subset Rn$

Rn	0	0	0
Ge	-2.010524	-0.653259	2.767248
Ge	2.010524	2.767248	0.653259
Ge	2.010524	-2.767248	-0.653259
Ge	2.010524	-0.653259	2.767248
Ge	0	-3.420507	0.653259
Ge	-1.242572	1.710253	2.767248
Ge	3.253095	1.056995	-0.653259
Ge	0	3.420507	-0.653259
Ge	1.242572	-1.710253	-2.767248
Ge	-3.253095	-1.056995	0.653259
Ge	2.010524	0.653259	-2.767248
Ge	-2.010524	-2.767248	-0.653259
Ge	-2.010524	2.767248	0.653259
Ge	-2.010524	0.653259	-2.767248
Ge	-1.242572	-1.710253	-2.767248
Ge	-3.253095	1.056995	-0.653259
Ge	0	2.11399	-2.767248
Ge	3.253095	-1.056995	0.653259
Ge	0	-2.11399	2.767248

Ge	1.242572	1.710253	2.767248
H	0	4.936289	-0.942747
H	1.793212	-2.468145	-3.993542
H	-4.69469	-1.525397	0.942747
H	2.901478	0.942747	-3.993542
H	-2.901478	-3.993542	-0.942747
H	-2.901478	3.993542	0.942747
H	-2.901478	0.942747	-3.993542
H	-1.793212	-2.468145	-3.993542
H	-4.69469	1.525397	-0.942747
H	0	3.050794	-3.993542
H	4.69469	1.525397	-0.942747
H	0	-4.936289	0.942747
H	-1.793212	2.468145	3.993542
H	-2.901478	-0.942747	3.993542
H	2.901478	3.993542	0.942747
H	2.901478	-3.993542	-0.942747
H	2.901478	-0.942747	3.993542
H	0	-3.050794	3.993542
H	1.793212	2.468145	3.993542
H	4.69469	-1.525397	0.942747

$Sn_{20}H_{20}$

Sn	-2.269448	-0.737388	3.123627
Sn	2.269448	3.123627	0.737388
Sn	2.269448	-3.123627	-0.737388
Sn	2.269448	-0.737388	3.123627
Sn	0	-3.861016	0.737388
Sn	-1.402596	1.930508	3.123627
Sn	3.672044	1.193119	-0.737388
Sn	0	3.861016	-0.737388

Sn	1.402596	-1.930508	-3.123627
Sn	-3.672044	-1.193119	0.737388
Sn	2.269448	0.737388	-3.123627
Sn	-2.269448	-3.123627	-0.737388
Sn	-2.269448	3.123627	0.737388
Sn	-2.269448	0.737388	-3.123627
Sn	-1.402596	-1.930508	-3.123627
Sn	-3.672044	1.193119	-0.737388
Sn	0	2.386239	-3.123627
Sn	3.672044	-1.193119	0.737388
Sn	0	-2.386239	3.123627
Sn	1.402596	1.930508	3.123627
H	0	5.549564	-1.059872
H	2.015997	-2.774782	-4.489691
H	-5.277949	-1.714909	1.059872
H	3.261952	1.059872	-4.489691
H	-3.261952	-4.489691	-1.059872
H	-3.261952	4.489691	1.059872
H	-3.261952	1.059872	-4.489691
H	-2.015997	-2.774782	-4.489691
H	-5.277949	1.714909	-1.059872
H	0	3.429819	-4.489691
H	5.277949	1.714909	-1.059872
H	0	-5.549564	1.059872
H	-2.015997	2.774782	4.489691
H	-3.261952	-1.059872	4.489691
H	3.261952	4.489691	1.059872
H	3.261952	-4.489691	-1.059872
H	3.261952	-1.059872	4.489691
H	0	-3.429819	4.489691
H	2.015997	2.774782	4.489691

H	5.277949	-1.714909	1.059872

$Sn_{20}H_{20} \subset He$

He	0	0	0
Sn	-2.269819	-0.737509	3.124137
Sn	2.269819	3.124137	0.737509
Sn	2.269819	-3.124137	-0.737509
Sn	2.269819	-0.737509	3.124137
Sn	0	-3.861646	0.737509
Sn	-1.402825	1.930823	3.124137
Sn	3.672643	1.193314	-0.737509
Sn	0	3.861646	-0.737509
Sn	1.402825	-1.930823	-3.124137
Sn	-3.672643	-1.193314	0.737509
Sn	2.269819	0.737509	-3.124137
Sn	-2.269819	-3.124137	-0.737509
Sn	-2.269819	3.124137	0.737509
Sn	-2.269819	0.737509	-3.124137
Sn	-1.402825	-1.930823	-3.124137
Sn	-3.672643	1.193314	-0.737509
Sn	0	2.386628	-3.124137
Sn	3.672643	-1.193314	0.737509
Sn	0	-2.386628	3.124137
Sn	1.402825	1.930823	3.124137
H	0	5.549075	-1.059779
H	2.01582	-2.774538	-4.489296
H	-5.277484	-1.714759	1.059779
H	3.261665	1.059779	-4.489296
H	-3.261665	-4.489296	-1.059779
H	-3.261665	4.489296	1.059779

H	-3.261665	1.059779	-4.489296
H	-2.01582	-2.774538	-4.489296
H	-5.277484	1.714759	-1.059779
H	0	3.429517	-4.489296
H	5.277484	1.714759	-1.059779
H	0	-5.549075	1.059779
H	-2.01582	2.774538	4.489296
H	-3.261665	-1.059779	4.489296
H	3.261665	4.489296	1.059779
H	3.261665	-4.489296	-1.059779
H	3.261665	-1.059779	4.489296
H	0	-3.429517	4.489296
H	2.01582	2.774538	4.489296
H	5.277484	-1.714759	1.059779

$Sn_{20}H_{20} \subset Ne$

Ne	0	0	0
Sn	-2.269119	-0.737281	3.123174
Sn	2.269119	3.123174	0.737281
Sn	2.269119	-3.123174	-0.737281
Sn	2.269119	-0.737281	3.123174
Sn	0	-3.860456	0.737281
Sn	-1.402393	1.930228	3.123174
Sn	3.671511	1.192946	-0.737281
Sn	0	3.860456	-0.737281
Sn	1.402393	-1.930228	-3.123174
Sn	-3.671511	-1.192946	0.737281
Sn	2.269119	0.737281	-3.123174
Sn	-2.269119	-3.123174	-0.737281
Sn	-2.269119	3.123174	0.737281

Sn	-2.269119	0.737281	-3.123174
Sn	-1.402393	-1.930228	-3.123174
Sn	-3.671511	1.192946	-0.737281
Sn	0	2.385893	-3.123174
Sn	3.671511	-1.192946	0.737281
Sn	0	-2.385893	3.123174
Sn	1.402393	1.930228	3.123174
H	0	5.547762	-1.059528
H	2.015343	-2.773881	-4.488234
H	-5.276236	-1.714353	1.059528
H	3.260893	1.059528	-4.488234
H	-3.260893	-4.488234	-1.059528
H	-3.260893	4.488234	1.059528
H	-3.260893	1.059528	-4.488234
H	-2.015343	-2.773881	-4.488234
H	-5.276236	1.714353	-1.059528
H	0	3.428706	-4.488234
H	5.276236	1.714353	-1.059528
H	0	-5.547762	1.059528
H	-2.015343	2.773881	4.488234
H	-3.260893	-1.059528	4.488234
H	3.260893	4.488234	1.059528
H	3.260893	-4.488234	-1.059528
H	3.260893	-1.059528	4.488234
H	0	-3.428706	4.488234
H	2.015343	2.773881	4.488234
H	5.276236	-1.714353	1.059528

$Sn_{20}H_{20} \subset Ar$

Ar	0	0	0

Sn	-2.272993	-0.73854	3.128506
Sn	2.272993	3.128506	0.73854
Sn	2.272993	-3.128506	-0.73854
Sn	2.272993	-0.73854	3.128506
Sn	0	-3.867047	0.73854
Sn	-1.404787	1.933523	3.128506
Sn	3.67778	1.194983	-0.73854
Sn	0	3.867047	-0.73854
Sn	1.404787	-1.933523	-3.128506
Sn	-3.67778	-1.194983	0.73854
Sn	2.272993	0.73854	-3.128506
Sn	-2.272993	-3.128506	-0.73854
Sn	-2.272993	3.128506	0.73854
Sn	-2.272993	0.73854	-3.128506
Sn	-1.404787	-1.933523	-3.128506
Sn	-3.67778	1.194983	-0.73854
Sn	0	2.389966	-3.128506
Sn	3.67778	-1.194983	0.73854
Sn	0	-2.389966	3.128506
Sn	1.404787	1.933523	3.128506
H	0	5.554537	-1.060822
H	2.017804	-2.777269	-4.493715
H	-5.282679	-1.716446	1.060822
H	3.264875	1.060822	-4.493715
H	-3.264875	-4.493715	-1.060822
H	-3.264875	4.493715	1.060822
H	-3.264875	1.060822	-4.493715
H	-2.017804	-2.777269	-4.493715
H	-5.282679	1.716446	-1.060822
H	0	3.432893	-4.493715
H	5.282679	1.716446	-1.060822

H	0	-5.554537	1.060822
H	-2.017804	2.777269	4.493715
H	-3.264875	-1.060822	4.493715
H	3.264875	4.493715	1.060822
H	3.264875	-4.493715	-1.060822
H	3.264875	-1.060822	4.493715
H	0	-3.432893	4.493715
H	2.017804	2.777269	4.493715
H	5.282679	-1.716446	1.060822

$Sn_{20}H_{20} \subset Kr$

Kr	0	0	0
Sn	-2.276959	-0.739829	3.133965
Sn	2.276959	3.133965	0.739829
Sn	2.276959	-3.133965	-0.739829
Sn	2.276959	-0.739829	3.133965
Sn	0	-3.873793	0.739829
Sn	-1.407238	1.936897	3.133965
Sn	3.684196	1.197068	-0.739829
Sn	0	3.873793	-0.739829
Sn	1.407238	-1.936897	-3.133965
Sn	-3.684196	-1.197068	0.739829
Sn	2.276959	0.739829	-3.133965
Sn	-2.276959	-3.133965	-0.739829
Sn	-2.276959	3.133965	0.739829
Sn	-2.276959	0.739829	-3.133965
Sn	-1.407238	-1.936897	-3.133965
Sn	-3.684196	1.197068	-0.739829
Sn	0	2.394136	-3.133965
Sn	3.684196	-1.197068	0.739829

Sn	0	-2.394136	3.133965
Sn	1.407238	1.936897	3.133965
H	0	5.561549	-1.062161
H	2.020351	-2.780774	-4.499387
H	-5.289347	-1.718613	1.062161
H	3.268996	1.062161	-4.499387
H	-3.268996	-4.499387	-1.062161
H	-3.268996	4.499387	1.062161
H	-3.268996	1.062161	-4.499387
H	-2.020351	-2.780774	-4.499387
H	-5.289347	1.718613	-1.062161
H	0	3.437226	-4.499387
H	5.289347	1.718613	-1.062161
H	0	-5.561549	1.062161
H	-2.020351	2.780774	4.499387
H	-3.268996	-1.062161	4.499387
H	3.268996	4.499387	1.062161
H	3.268996	-4.499387	-1.062161
H	3.268996	-1.062161	4.499387
H	0	-3.437226	4.499387
H	2.020351	2.780774	4.499387
H	5.289347	-1.718613	1.062161

$Sn_{20}H_{20} \subset Xe$

Xe	0	0	0
Sn	-2.28302	-0.741798	3.142308
Sn	2.28302	3.142308	0.741798
Sn	2.28302	-3.142308	-0.741798
Sn	2.28302	-0.741798	3.142308
Sn	0	-3.884106	0.741798

Sn	-1.410984	1.942053	3.142308
Sn	3.694004	1.200255	-0.741798
Sn	0	3.884106	-0.741798
Sn	1.410984	-1.942053	-3.142308
Sn	-3.694004	-1.200255	0.741798
Sn	2.28302	0.741798	-3.142308
Sn	-2.28302	-3.142308	-0.741798
Sn	-2.28302	3.142308	0.741798
Sn	-2.28302	0.741798	-3.142308
Sn	-1.410984	-1.942053	-3.142308
Sn	-3.694004	1.200255	-0.741798
Sn	0	2.40051	-3.142308
Sn	3.694004	-1.200255	0.741798
Sn	0	-2.40051	3.142308
Sn	1.410984	1.942053	3.142308
H	0	5.572413	-1.064236
H	2.024298	-2.786207	-4.508177
H	-5.29968	-1.72197	1.064236
H	3.275382	1.064236	-4.508177
H	-3.275382	-4.508177	-1.064236
H	-3.275382	4.508177	1.064236
H	-3.275382	1.064236	-4.508177
H	-2.024298	-2.786207	-4.508177
H	-5.29968	1.72197	-1.064236
H	0	3.443941	-4.508177
H	5.29968	1.72197	-1.064236
H	0	-5.572413	1.064236
H	-2.024298	2.786207	4.508177
H	-3.275382	-1.064236	4.508177
H	3.275382	4.508177	1.064236
H	3.275382	-4.508177	-1.064236

H	3.275382	-1.064236	4.508177
H	0	-3.443941	4.508177
H	2.024298	2.786207	4.508177
H	5.29968	-1.72197	1.064236

$Sn_{20}H_{20} \subset Rn$

Rn	0	0	0
Sn	-2.285855	-0.742719	3.14621
Sn	2.285855	3.14621	0.742719
Sn	2.285855	-3.14621	-0.742719
Sn	2.285855	-0.742719	3.14621
Sn	0	-3.888929	0.742719
Sn	-1.412736	1.944465	3.14621
Sn	3.698591	1.201745	-0.742719
Sn	0	3.888929	-0.742719
Sn	1.412736	-1.944465	-3.14621
Sn	-3.698591	-1.201745	0.742719
Sn	2.285855	0.742719	-3.14621
Sn	-2.285855	-3.14621	-0.742719
Sn	-2.285855	3.14621	0.742719
Sn	-2.285855	0.742719	-3.14621
Sn	-1.412736	-1.944465	-3.14621
Sn	-3.698591	1.201745	-0.742719
Sn	0	2.40349	-3.14621
Sn	3.698591	-1.201745	0.742719
Sn	0	-2.40349	3.14621
Sn	1.412736	1.944465	3.14621
H	0	5.577538	-1.065215
H	2.026159	-2.788769	-4.512323
H	-5.304554	-1.723554	1.065215

H	3.278395	1.065215	-4.512323
H	-3.278395	-4.512323	-1.065215
H	-3.278395	4.512323	1.065215
H	-3.278395	1.065215	-4.512323
H	-2.026159	-2.788769	-4.512323
H	-5.304554	1.723554	-1.065215
H	0	3.447108	-4.512323
H	5.304554	1.723554	-1.065215
H	0	-5.577538	1.065215
H	-2.026159	2.788769	4.512323
H	-3.278395	-1.065215	4.512323
H	3.278395	4.512323	1.065215
H	3.278395	-4.512323	-1.065215
H	3.278395	-1.065215	4.512323
H	0	-3.447108	4.512323
H	2.026159	2.788769	4.512323
H	5.304554	-1.723554	1.065215

$Pb_{20}H_{20}$

Pb	-2.37353	-0.771207	3.266884
Pb	2.37353	3.266884	0.771207
Pb	2.37353	-3.266884	-0.771207
Pb	2.37353	-0.771207	3.266884
Pb	0	-4.038091	0.771207
Pb	-1.466922	2.019045	3.266884
Pb	3.840453	1.247839	-0.771207
Pb	0	4.038091	-0.771207
Pb	1.466922	-2.019045	-3.266884
Pb	-3.840453	-1.247839	0.771207
Pb	2.37353	0.771207	-3.266884
Pb	-2.37353	-3.266884	-0.771207

Pb	-2.37353	3.266884	0.771207
Pb	-2.37353	0.771207	-3.266884
Pb	-1.466922	-2.019045	-3.266884
Pb	-3.840453	1.247839	-0.771207
Pb	0	2.495677	-3.266884
Pb	3.840453	-1.247839	0.771207
Pb	0	-2.495677	3.266884
Pb	1.466922	2.019045	3.266884
H	0	5.788459	-1.105497
H	2.102781	-2.894229	-4.682962
H	-5.505152	-1.788732	1.105497
H	3.402371	1.105497	-4.682962
H	-3.402371	-4.682962	-1.105497
H	-3.402371	4.682962	1.105497
H	-3.402371	1.105497	-4.682962
H	-2.102781	-2.894229	-4.682962
H	-5.505152	1.788732	-1.105497
H	0	3.577464	-4.682962
H	5.505152	1.788732	-1.105497
H	0	-5.788459	1.105497
H	-2.102781	2.894229	4.682962
H	-3.402371	-1.105497	4.682962
H	3.402371	4.682962	1.105497
H	3.402371	-4.682962	-1.105497
H	3.402371	-1.105497	4.682962
H	0	-3.577464	4.682962
H	2.102781	2.894229	4.682962
H	5.505152	-1.788732	1.105497

$Pb_{20}H_{20} \subset He$

He	0	0	0
Pb	-2.373227	-0.771108	3.266467
Pb	2.373227	3.266467	0.771108
Pb	2.373227	-3.266467	-0.771108
Pb	2.373227	-0.771108	3.266467
Pb	0	-4.037576	0.771108
Pb	-1.466735	2.018788	3.266467
Pb	3.839963	1.247679	-0.771108
Pb	0	4.037576	-0.771108
Pb	1.466735	-2.018788	-3.266467
Pb	-3.839963	-1.247679	0.771108
Pb	2.373227	0.771108	-3.266467
Pb	-2.373227	-3.266467	-0.771108
Pb	-2.373227	3.266467	0.771108
Pb	-2.373227	0.771108	-3.266467
Pb	-1.466735	-2.018788	-3.266467
Pb	-3.839963	1.247679	-0.771108
Pb	0	2.495359	-3.266467
Pb	3.839963	-1.247679	0.771108
Pb	0	-2.495359	3.266467
Pb	1.466735	2.018788	3.266467
H	0	5.78868	-1.105539
H	2.102861	-2.89434	-4.68314
H	-5.505362	-1.7888	1.105539
H	3.402501	1.105539	-4.68314
H	-3.402501	-4.68314	-1.105539
H	-3.402501	4.68314	1.105539
H	-3.402501	1.105539	-4.68314
H	-2.102861	-2.89434	-4.68314
H	-5.505362	1.7888	-1.105539
H	0	3.577601	-4.68314

H	5.505362	1.7888	-1.105539
H	0	-5.78868	1.105539
H	-2.102861	2.89434	4.68314
H	-3.402501	-1.105539	4.68314
H	3.402501	4.68314	1.105539
H	3.402501	-4.68314	-1.105539
H	3.402501	-1.105539	4.68314
H	0	-3.577601	4.68314
H	2.102861	2.89434	4.68314
H	5.505362	-1.7888	1.105539

$Pb_{20}H_{20} \subset Ne$

Ne	0	0	0
Pb	-2.372402	-0.77084	3.265331
Pb	2.372402	3.265331	0.77084
Pb	2.372402	-3.265331	-0.77084
Pb	2.372402	-0.77084	3.265331
Pb	0	-4.036171	0.77084
Pb	-1.466225	2.018086	3.265331
Pb	3.838627	1.247246	-0.77084
Pb	0	4.036171	-0.77084
Pb	1.466225	-2.018086	-3.265331
Pb	-3.838627	-1.247246	0.77084
Pb	2.372402	0.77084	-3.265331
Pb	-2.372402	-3.265331	-0.77084
Pb	-2.372402	3.265331	0.77084
Pb	-2.372402	0.77084	-3.265331
Pb	-1.466225	-2.018086	-3.265331
Pb	-3.838627	1.247246	-0.77084
Pb	0	2.494491	-3.265331

Pb	3.838627	-1.247246	0.77084
Pb	0	-2.494491	3.265331
Pb	1.466225	2.018086	3.265331
H	0	5.787166	-1.10525
H	2.102311	-2.893583	-4.681916
H	-5.503922	-1.788333	1.10525
H	3.401611	1.10525	-4.681916
H	-3.401611	-4.681916	-1.10525
H	-3.401611	4.681916	1.10525
H	-3.401611	1.10525	-4.681916
H	-2.102311	-2.893583	-4.681916
H	-5.503922	1.788333	-1.10525
H	0	3.576666	-4.681916
H	5.503922	1.788333	-1.10525
H	0	-5.787166	1.10525
H	-2.102311	2.893583	4.681916
H	-3.401611	-1.10525	4.681916
H	3.401611	4.681916	1.10525
H	3.401611	-4.681916	-1.10525
H	3.401611	-1.10525	4.681916
H	0	-3.576666	4.681916
H	2.102311	2.893583	4.681916
H	5.503922	-1.788333	1.10525

$Pb_{20}H_{20} \subset Ar$

Ar	0	0	0
Pb	-2.375612	-0.771883	3.269749
Pb	2.375612	3.269749	0.771883
Pb	2.375612	-3.269749	-0.771883
Pb	2.375612	-0.771883	3.269749

Pb	0	-4.041633	0.771883
Pb	-1.468209	2.020816	3.269749
Pb	3.843821	1.248933	-0.771883
Pb	0	4.041633	-0.771883
Pb	1.468209	-2.020816	-3.269749
Pb	-3.843821	-1.248933	0.771883
Pb	2.375612	0.771883	-3.269749
Pb	-2.375612	-3.269749	-0.771883
Pb	-2.375612	3.269749	0.771883
Pb	-2.375612	0.771883	-3.269749
Pb	-1.468209	-2.020816	-3.269749
Pb	-3.843821	1.248933	-0.771883
Pb	0	2.497866	-3.269749
Pb	3.843821	-1.248933	0.771883
Pb	0	-2.497866	3.269749
Pb	1.468209	2.020816	3.269749
H	0	5.793188	-1.1064
H	2.104499	-2.896594	-4.686787
H	-5.509649	-1.790193	1.1064
H	3.40515	1.1064	-4.686787
H	-3.40515	-4.686787	-1.1064
H	-3.40515	4.686787	1.1064
H	-3.40515	1.1064	-4.686787
H	-2.104499	-2.896594	-4.686787
H	-5.509649	1.790193	-1.1064
H	0	3.580387	-4.686787
H	5.509649	1.790193	-1.1064
H	0	-5.793188	1.1064
H	-2.104499	2.896594	4.686787
H	-3.40515	-1.1064	4.686787
H	3.40515	4.686787	1.1064

H	3.40515	-4.686787	-1.1064
H	3.40515	-1.1064	4.686787
H	0	-3.580387	4.686787
H	2.104499	2.896594	4.686787
H	5.509649	-1.790193	1.1064

$Pb_{20}H_{20} \subset Kr$

Kr	0	0	0
Pb	-2.378474	-0.772813	3.273688
Pb	2.378474	3.273688	0.772813
Pb	2.378474	-3.273688	-0.772813
Pb	2.378474	-0.772813	3.273688
Pb	0	-4.046501	0.772813
Pb	-1.469978	2.023251	3.273688
Pb	3.848451	1.250438	-0.772813
Pb	0	4.046501	-0.772813
Pb	1.469978	-2.023251	-3.273688
Pb	-3.848451	-1.250438	0.772813
Pb	2.378474	0.772813	-3.273688
Pb	-2.378474	-3.273688	-0.772813
Pb	-2.378474	3.273688	0.772813
Pb	-2.378474	0.772813	-3.273688
Pb	-1.469978	-2.023251	-3.273688
Pb	-3.848451	1.250438	-0.772813
Pb	0	2.500875	-3.273688
Pb	3.848451	-1.250438	0.772813
Pb	0	-2.500875	3.273688
Pb	1.469978	2.023251	3.273688
H	0	5.797334	-1.107192
H	2.106005	-2.898667	-4.690142

H	-5.513593	-1.791475	1.107192
H	3.407588	1.107192	-4.690142
H	-3.407588	-4.690142	-1.107192
H	-3.407588	4.690142	1.107192
H	-3.407588	1.107192	-4.690142
H	-2.106005	-2.898667	-4.690142
H	-5.513593	1.791475	-1.107192
H	0	3.58295	-4.690142
H	5.513593	1.791475	-1.107192
H	0	-5.797334	1.107192
H	-2.106005	2.898667	4.690142
H	-3.407588	-1.107192	4.690142
H	3.407588	4.690142	1.107192
H	3.407588	-4.690142	-1.107192
H	3.407588	-1.107192	4.690142
H	0	-3.58295	4.690142
H	2.106005	2.898667	4.690142
H	5.513593	-1.791475	1.107192

$Pb_{20}H_{20} \subset Xe$

Xe	0	0	0
Pb	-2.385109	-0.774969	3.282821
Pb	2.385109	3.282821	0.774969
Pb	2.385109	-3.282821	-0.774969
Pb	2.385109	-0.774969	3.282821
Pb	0	-4.05779	0.774969
Pb	-1.474078	2.028895	3.282821
Pb	3.859187	1.253926	-0.774969
Pb	0	4.05779	-0.774969
Pb	1.474078	-2.028895	-3.282821

Pb	-3.859187	-1.253926	0.774969
Pb	2.385109	0.774969	-3.282821
Pb	-2.385109	-3.282821	-0.774969
Pb	-2.385109	3.282821	0.774969
Pb	-2.385109	0.774969	-3.282821
Pb	-1.474078	-2.028895	-3.282821
Pb	-3.859187	1.253926	-0.774969
Pb	0	2.507852	-3.282821
Pb	3.859187	-1.253926	0.774969
Pb	0	-2.507852	3.282821
Pb	1.474078	2.028895	3.282821
H	0	5.809412	-1.109499
H	2.110393	-2.904706	-4.699913
H	-5.52508	-1.795207	1.109499
H	3.414687	1.109499	-4.699913
H	-3.414687	-4.699913	-1.109499
H	-3.414687	4.699913	1.109499
H	-3.414687	1.109499	-4.699913
H	-2.110393	-2.904706	-4.699913
H	-5.52508	1.795207	-1.109499
H	0	3.590414	-4.699913
H	5.52508	1.795207	-1.109499
H	0	-5.809412	1.109499
H	-2.110393	2.904706	4.699913
H	-3.414687	-1.109499	4.699913
H	3.414687	4.699913	1.109499
H	3.414687	-4.699913	-1.109499
H	3.414687	-1.109499	4.699913
H	0	-3.590414	4.699913
H	2.110393	2.904706	4.699913
H	5.52508	-1.795207	1.109499

$Pb_{20}H_{20} \subset Rn$

Rn	0	0	0
Pb	-2.388697	-0.776135	3.28776
Pb	2.388697	3.28776	0.776135
Pb	2.388697	-3.28776	-0.776135
Pb	2.388697	-0.776135	3.28776
Pb	0	-4.063895	0.776135
Pb	-1.476296	2.031947	3.28776
Pb	3.864994	1.255813	-0.776135
Pb	0	4.063895	-0.776135
Pb	1.476296	-2.031947	-3.28776
Pb	-3.864994	-1.255813	0.776135
Pb	2.388697	0.776135	-3.28776
Pb	-2.388697	-3.28776	-0.776135
Pb	-2.388697	3.28776	0.776135
Pb	-2.388697	0.776135	-3.28776
Pb	-1.476296	-2.031947	-3.28776
Pb	-3.864994	1.255813	-0.776135
Pb	0	2.511625	-3.28776
Pb	3.864994	-1.255813	0.776135
Pb	0	-2.511625	3.28776
Pb	1.476296	2.031947	3.28776
H	0	5.815973	-1.110752
H	2.112776	-2.907987	-4.705221
H	-5.531319	-1.797235	1.110752
H	3.418543	1.110752	-4.705221
H	-3.418543	-4.705221	-1.110752
H	-3.418543	4.705221	1.110752
H	-3.418543	1.110752	-4.705221

H	-2.112776	-2.907987	-4.705221
H	-5.531319	1.797235	-1.110752
H	0	3.594469	-4.705221
H	5.531319	1.797235	-1.110752
H	0	-5.815973	1.110752
H	-2.112776	2.907987	4.705221
H	-3.418543	-1.110752	4.705221
H	3.418543	4.705221	1.110752
H	3.418543	-4.705221	-1.110752
H	3.418543	-1.110752	4.705221
H	0	-3.594469	4.705221
H	2.112776	2.907987	4.705221
H	5.531319	-1.797235	1.110752

N_{20}

N	-1.192808	-0.387567	1.641759
N	0	-1.254192	1.641759
N	1.192808	-0.387567	1.641759
N	0.737196	1.014663	1.641759
N	-0.737196	1.014663	1.641759
N	0	-2.029325	0.387567
N	-1.192808	-1.641759	-0.387567
N	-1.930003	-0.627096	0.387567
N	1.192808	-1.641759	-0.387567
N	1.930003	-0.627096	0.387567
N	1.930003	0.627096	-0.387567
N	1.192808	1.641759	0.387567
N	0	2.029325	-0.387567
N	-1.192808	1.641759	0.387567
N	-1.930003	0.627096	-0.387567
N	0.737196	-1.014663	-1.641759

N	1.192808	0.387567	-1.641759
N	0	1.254192	-1.641759
N	-1.192808	0.387567	-1.641759
N	-0.737196	-1.014663	-1.641759

$N_{20} \subset He$

N	-1.192808	-0.387567	1.641759
N	0	-1.254192	1.641759
N	1.192808	-0.387567	1.641759
N	0.737196	1.014663	1.641759
N	-0.737196	1.014663	1.641759
N	0	-2.029325	0.387567
N	-1.192808	-1.641759	-0.387567
N	-1.930003	-0.627096	0.387567
N	1.192808	-1.641759	-0.387567
N	1.930003	-0.627096	0.387567
N	1.930003	0.627096	-0.387567
N	1.192808	1.641759	0.387567
N	0	2.029325	-0.387567
N	-1.192808	1.641759	0.387567
N	-1.930003	0.627096	-0.387567
N	0.737196	-1.014663	-1.641759
N	1.192808	0.387567	-1.641759
N	0	1.254192	-1.641759
N	-1.192808	0.387567	-1.641759
N	-0.737196	-1.014663	-1.641759
He	0	0	0

$N_{20} \subset Ne$

N	-1.215643	-0.394986	1.673189

N	0	-1.278203	1.673189
N	1.215643	-0.394986	1.673189
N	0.751309	1.034088	1.673189
N	-0.751309	1.034088	1.673189
N	0	-2.068175	0.394986
N	-1.215643	-1.673189	-0.394986
N	-1.966952	-0.639101	0.394986
N	1.215643	-1.673189	-0.394986
N	1.966952	-0.639101	0.394986
N	1.966952	0.639101	-0.394986
N	1.215643	1.673189	0.394986
N	0	2.068175	-0.394986
N	-1.215643	1.673189	0.394986
N	-1.966952	0.639101	-0.394986
N	0.751309	-1.034088	-1.673189
N	1.215643	0.394986	-1.673189
N	0	1.278203	-1.673189
N	-1.215643	0.394986	-1.673189
N	-0.751309	-1.034088	-1.673189
Ne	0	0	0

$N_{20} \subset Ar$

N	-1.274956	-0.414258	1.754827
N	0	-1.340568	1.754827
N	1.274956	-0.414258	1.754827
N	0.787966	1.084543	1.754827
N	-0.787966	1.084543	1.754827
N	0	-2.169085	0.414258
N	-1.274956	-1.754827	-0.414258
N	-2.062923	-0.670284	0.414258

N	1.274956	-1.754827	-0.414258
N	2.062923	-0.670284	0.414258
N	2.062923	0.670284	-0.414258
N	1.274956	1.754827	0.414258
N	0	2.169085	-0.414258
N	-1.274956	1.754827	0.414258
N	-2.062923	0.670284	-0.414258
N	0.787966	-1.084543	-1.754827
N	1.274956	0.414258	-1.754827
N	0	1.340568	-1.754827
N	-1.274956	0.414258	-1.754827
N	-0.787966	-1.084543	-1.754827
Ar	0	0	0

$N_{20} \subset Kr$

N	-1.310337	-0.425754	1.803524
N	0	-1.37777	1.803524
N	1.310337	-0.425754	1.803524
N	0.809833	1.114639	1.803524
N	-0.809833	1.114639	1.803524
N	0	-2.229278	0.425754
N	-1.310337	-1.803524	-0.425754
N	-2.12017	-0.688885	0.425754
N	1.310337	-1.803524	-0.425754
N	2.12017	-0.688885	0.425754
N	2.12017	0.688885	-0.425754
N	1.310337	1.803524	0.425754
N	0	2.229278	-0.425754
N	-1.310337	1.803524	0.425754
N	-2.12017	0.688885	-0.425754

N	0.809833	-1.114639	-1.803524
N	1.310337	0.425754	-1.803524
N	0	1.37777	-1.803524
N	-1.310337	0.425754	-1.803524
N	-0.809833	-1.114639	-1.803524
Kr	0	0	0

$N_{20} \subset Xe$

N	-1.346024	-0.43735	1.852644
N	0	-1.415294	1.852644
N	1.346024	-0.43735	1.852644
N	0.831889	1.144997	1.852644
N	-0.831889	1.144997	1.852644
N	0	-2.289994	0.43735
N	-1.346024	-1.852644	-0.43735
N	-2.177913	-0.707647	0.43735
N	1.346024	-1.852644	-0.43735
N	2.177913	-0.707647	0.43735
N	2.177913	0.707647	-0.43735
N	1.346024	1.852644	0.43735
N	0	2.289994	-0.43735
N	-1.346024	1.852644	0.43735
N	-2.177913	0.707647	-0.43735
N	0.831889	-1.144997	-1.852644
N	1.346024	0.43735	-1.852644
N	0	1.415294	-1.852644
N	-1.346024	0.43735	-1.852644
N	-0.831889	-1.144997	-1.852644
Xe	0	0	0

$N_{20} \subset Rn$

N	-1.358866	-0.441522	1.870318
N	0	-1.428796	1.870318
N	1.358866	-0.441522	1.870318
N	0.839825	1.15592	1.870318
N	-0.839825	1.15592	1.870318
N	0	-2.31184	0.441522
N	-1.358866	-1.870318	-0.441522
N	-2.198691	-0.714398	0.441522
N	1.358866	-1.870318	-0.441522
N	2.198691	-0.714398	0.441522
N	2.198691	0.714398	-0.441522
N	1.358866	1.870318	0.441522
N	0	2.31184	-0.441522
N	-1.358866	1.870318	0.441522
N	-2.198691	0.714398	-0.441522
N	0.839825	-1.15592	-1.870318
N	1.358866	0.441522	-1.870318
N	0	1.428796	-1.870318
N	-1.358866	0.441522	-1.870318
N	-0.839825	-1.15592	-1.870318
Rn	0	0	0

P_{20}

P	-1.823965	-0.592642	2.510472
P	0	-1.91783	2.510472
P	1.823965	-0.592642	2.510472
P	1.127272	1.551557	2.510472
P	-1.127272	1.551557	2.510472
P	0	-3.103114	0.592642

P	-1.823965	-2.510472	-0.592642
P	-2.951237	-0.958915	0.592642
P	1.823965	-2.510472	-0.592642
P	2.951237	-0.958915	0.592642
P	2.951237	0.958915	-0.592642
P	1.823965	2.510472	0.592642
P	0	3.103114	-0.592642
P	-1.823965	2.510472	0.592642
P	-2.951237	0.958915	-0.592642
P	1.127272	-1.551557	-2.510472
P	1.823965	0.592642	-2.510472
P	0	1.91783	-2.510472
P	-1.823965	0.592642	-2.510472
P	-1.127272	-1.551557	-2.510472

$P_{20} \subset He$

P	-1.826071	-0.593327	2.513372
P	0	-1.920045	2.513372
P	1.826071	-0.593327	2.513372
P	1.128574	1.553349	2.513372
P	-1.128574	1.553349	2.513372
P	0	-3.106698	0.593327
P	-1.826071	-2.513372	-0.593327
P	-2.954646	-0.960023	0.593327
P	1.826071	-2.513372	-0.593327
P	2.954646	-0.960023	0.593327
P	2.954646	0.960023	-0.593327
P	1.826071	2.513372	0.593327
P	0	3.106698	-0.593327
P	-1.826071	2.513372	0.593327

P	-2.954646	0.960023	-0.593327
P	1.128574	-1.553349	-2.513372
P	1.826071	0.593327	-2.513372
P	0	1.920045	-2.513372
P	-1.826071	0.593327	-2.513372
P	-1.128574	-1.553349	-2.513372
He	0	0	0

$P_{20} \subset Ne$

P	-1.828203	-0.594019	2.516306
P	0	-1.922287	2.516306
P	1.828203	-0.594019	2.516306
P	1.129892	1.555163	2.516306
P	-1.129892	1.555163	2.516306
P	0	-3.110325	0.594019
P	-1.828203	-2.516306	-0.594019
P	-2.958095	-0.961143	0.594019
P	1.828203	-2.516306	-0.594019
P	2.958095	-0.961143	0.594019
P	2.958095	0.961143	-0.594019
P	1.828203	2.516306	0.594019
P	0	3.110325	-0.594019
P	-1.828203	2.516306	0.594019
P	-2.958095	0.961143	-0.594019
P	1.129892	-1.555163	-2.516306
P	1.828203	0.594019	-2.516306
P	0	1.922287	-2.516306
P	-1.828203	0.594019	-2.516306
P	-1.129892	-1.555163	-2.516306
Ne	0	0	0

$P_{20} \subset Ar$

P	-1.843011	-0.598831	2.536687
P	0	-1.937856	2.536687
P	1.843011	-0.598831	2.536687
P	1.139043	1.567759	2.536687
P	-1.139043	1.567759	2.536687
P	0	-3.135518	0.598831
P	-1.843011	-2.536687	-0.598831
P	-2.982054	-0.968928	0.598831
P	1.843011	-2.536687	-0.598831
P	2.982054	-0.968928	0.598831
P	2.982054	0.968928	-0.598831
P	1.843011	2.536687	0.598831
P	0	3.135518	-0.598831
P	-1.843011	2.536687	0.598831
P	-2.982054	0.968928	-0.598831
P	1.139043	-1.567759	-2.536687
P	1.843011	0.598831	-2.536687
P	0	1.937856	-2.536687
P	-1.843011	0.598831	-2.536687
P	-1.139043	-1.567759	-2.536687
Ar	0	0	0

$P_{20} \subset Kr$

P	-1.851851	-0.601703	2.548854
P	0	-1.947151	2.548854
P	1.851851	-0.601703	2.548854
P	1.144507	1.575278	2.548854
P	-1.144507	1.575278	2.548854

P	0	-3.150557	0.601703
P	-1.851851	-2.548854	-0.601703
P	-2.996358	-0.973576	0.601703
P	1.851851	-2.548854	-0.601703
P	2.996358	-0.973576	0.601703
P	2.996358	0.973576	-0.601703
P	1.851851	2.548854	0.601703
P	0	3.150557	-0.601703
P	-1.851851	2.548854	0.601703
P	-2.996358	0.973576	-0.601703
P	1.144507	-1.575278	-2.548854
P	1.851851	0.601703	-2.548854
P	0	1.947151	-2.548854
P	-1.851851	0.601703	-2.548854
P	-1.144507	-1.575278	-2.548854
Kr	0	0	0

$P_{20} \subset Xe$

P	-1.866872	-0.606583	2.569529
P	0	-1.962945	2.569529
P	1.866872	-0.606583	2.569529
P	1.15379	1.588056	2.569529
P	-1.15379	1.588056	2.569529
P	0	-3.176112	0.606583
P	-1.866872	-2.569529	-0.606583
P	-3.020662	-0.981473	0.606583
P	1.866872	-2.569529	-0.606583
P	3.020662	-0.981473	0.606583
P	3.020662	0.981473	-0.606583
P	1.866872	2.569529	0.606583

P	0	3.176112	-0.606583
P	-1.866872	2.569529	0.606583
P	-3.020662	0.981473	-0.606583
P	1.15379	-1.588056	-2.569529
P	1.866872	0.606583	-2.569529
P	0	1.962945	-2.569529
P	-1.866872	0.606583	-2.569529
P	-1.15379	-1.588056	-2.569529
Xe	0	0	0

$P_{20} \subset Rn$

P	-1.875667	-0.609441	2.581635
P	0	-1.972193	2.581635
P	1.875667	-0.609441	2.581635
P	1.159226	1.595538	2.581635
P	-1.159226	1.595538	2.581635
P	0	-3.191076	0.609441
P	-1.875667	-2.581635	-0.609441
P	-3.034894	-0.986097	0.609441
P	1.875667	-2.581635	-0.609441
P	3.034894	-0.986097	0.609441
P	3.034894	0.986097	-0.609441
P	1.875667	2.581635	0.609441
P	0	3.191076	-0.609441
P	-1.875667	2.581635	0.609441
P	-3.034894	0.986097	-0.609441
P	1.159226	-1.595538	-2.581635
P	1.875667	0.609441	-2.581635
P	0	1.972193	-2.581635
P	-1.875667	0.609441	-2.581635

P	-1.159226	-1.595538	-2.581635
Rn	0	0	0

As_{20}

As	-1.987006	-0.645617	2.734879
As	0	-2.089262	2.734879
As	1.987006	-0.645617	2.734879
As	1.228037	1.690248	2.734879
As	-1.228037	1.690248	2.734879
As	0	-3.380496	0.645617
As	-1.987006	-2.734879	-0.645617
As	-3.215043	-1.044631	0.645617
As	1.987006	-2.734879	-0.645617
As	3.215043	-1.044631	0.645617
As	3.215043	1.044631	-0.645617
As	1.987006	2.734879	0.645617
As	0	3.380496	-0.645617
As	-1.987006	2.734879	0.645617
As	-3.215043	1.044631	-0.645617
As	1.228037	-1.690248	-2.734879
As	1.987006	0.645617	-2.734879
As	0	2.089262	-2.734879
As	-1.987006	0.645617	-2.734879
As	-1.228037	-1.690248	-2.734879

$As_{20} \subset He$

As	-1.987855	-0.645893	2.736047
As	0	-2.090154	2.736047
As	1.987855	-0.645893	2.736047
As	1.228562	1.69097	2.736047

As	-1.228562	1.69097	2.736047
As	0	-3.38194	0.645893
As	-1.987855	-2.736047	-0.645893
As	-3.216416	-1.045077	0.645893
As	1.987855	-2.736047	-0.645893
As	3.216416	-1.045077	0.645893
As	3.216416	1.045077	-0.645893
As	1.987855	2.736047	0.645893
As	0	3.38194	-0.645893
As	-1.987855	2.736047	0.645893
As	-3.216416	1.045077	-0.645893
As	1.228562	-1.69097	-2.736047
As	1.987855	0.645893	-2.736047
As	0	2.090154	-2.736047
As	-1.987855	0.645893	-2.736047
As	-1.228562	-1.69097	-2.736047
He	0	0	0

$As_{20} \subset Ne$

As	-1.989434	-0.646406	2.738222
As	0	-2.091815	2.738222
As	1.989434	-0.646406	2.738222
As	1.229538	1.692314	2.738222
As	-1.229538	1.692314	2.738222
As	0	-3.384628	0.646406
As	-1.989434	-2.738222	-0.646406
As	-3.218973	-1.045908	0.646406
As	1.989434	-2.738222	-0.646406
As	3.218973	-1.045908	0.646406
As	3.218973	1.045908	-0.646406

As	1.989434	2.738222	0.646406
As	0	3.384628	-0.646406
As	-1.989434	2.738222	0.646406
As	-3.218973	1.045908	-0.646406
As	1.229538	-1.692314	-2.738222
As	1.989434	0.646406	-2.738222
As	0	2.091815	-2.738222
As	-1.989434	0.646406	-2.738222
As	-1.229538	-1.692314	-2.738222
Ne	0	0	0

$As_{20} \subset Ar$

As	-1.998763	-0.649438	2.751062
As	0	-2.101624	2.751062
As	1.998763	-0.649438	2.751062
As	1.235304	1.70025	2.751062
As	-1.235304	1.70025	2.751062
As	0	-3.400499	0.649438
As	-1.998763	-2.751062	-0.649438
As	-3.234067	-1.050812	0.649438
As	1.998763	-2.751062	-0.649438
As	3.234067	-1.050812	0.649438
As	3.234067	1.050812	-0.649438
As	1.998763	2.751062	0.649438
As	0	3.400499	-0.649438
As	-1.998763	2.751062	0.649438
As	-3.234067	1.050812	-0.649438
As	1.235304	-1.70025	-2.751062
As	1.998763	0.649438	-2.751062
As	0	2.101624	-2.751062

As	-1.998763	0.649438	-2.751062
As	-1.235304	-1.70025	-2.751062
Ar	0	0	0

$As_{20} \subset Kr$

As	-2.006346	-0.651901	2.761498
As	0	-2.109597	2.761498
As	2.006346	-0.651901	2.761498
As	1.23999	1.7067	2.761498
As	-1.23999	1.7067	2.761498
As	0	-3.413399	0.651901
As	-2.006346	-2.761498	-0.651901
As	-3.246336	-1.054798	0.651901
As	2.006346	-2.761498	-0.651901
As	3.246336	-1.054798	0.651901
As	3.246336	1.054798	-0.651901
As	2.006346	2.761498	0.651901
As	0	3.413399	-0.651901
As	-2.006346	2.761498	0.651901
As	-3.246336	1.054798	-0.651901
As	1.23999	-1.7067	-2.761498
As	2.006346	0.651901	-2.761498
As	0	2.109597	-2.761498
As	-2.006346	0.651901	-2.761498
As	-1.23999	-1.7067	-2.761498
Kr	0	0	0

$As_{20} \subset Xe$

As	-2.018344	-0.6558	2.778013
As	0	-2.122213	2.778013

As	2.018344	-0.6558	2.778013
As	1.247405	1.716906	2.778013
As	-1.247405	1.716906	2.778013
As	0	-3.433813	0.6558
As	-2.018344	-2.778013	-0.6558
As	-3.26575	-1.061106	0.6558
As	2.018344	-2.778013	-0.6558
As	3.26575	-1.061106	0.6558
As	3.26575	1.061106	-0.6558
As	2.018344	2.778013	0.6558
As	0	3.433813	-0.6558
As	-2.018344	2.778013	0.6558
As	-3.26575	1.061106	-0.6558
As	1.247405	-1.716906	-2.778013
As	2.018344	0.6558	-2.778013
As	0	2.122213	-2.778013
As	-2.018344	0.6558	-2.778013
As	-1.247405	-1.716906	-2.778013
Xe	0	0	0

$As_{20} \subset Rn$

Rn	0	0	0
As	-2.025654	-0.658175	2.788073
As	0	-2.129898	2.788073
As	2.025654	-0.658175	2.788073
As	1.251923	1.723124	2.788073
As	-1.251923	1.723124	2.788073
As	0	-3.446248	0.658175
As	-2.025654	-2.788073	-0.658175
As	-3.277577	-1.064949	0.658175

As	2.025654	-2.788073	-0.658175
As	3.277577	-1.064949	0.658175
As	3.277577	1.064949	-0.658175
As	2.025654	2.788073	0.658175
As	0	3.446248	-0.658175
As	-2.025654	2.788073	0.658175
As	-3.277577	1.064949	-0.658175
As	1.251923	-1.723124	-2.788073
As	2.025654	0.658175	-2.788073
As	0	2.129898	-2.788073
As	-2.025654	0.658175	-2.788073
As	-1.251923	-1.723124	-2.788073

Sb_{20}

Sb	-2.296637	-0.746223	3.16105
Sb	0	-2.414827	3.16105
Sb	2.296637	-0.746223	3.16105
Sb	1.4194	1.953636	3.16105
Sb	-1.4194	1.953636	3.16105
Sb	0	-3.907273	0.746223
Sb	-2.296637	-3.16105	-0.746223
Sb	-3.716037	-1.207414	0.746223
Sb	2.296637	-3.16105	-0.746223
Sb	3.716037	-1.207414	0.746223
Sb	3.716037	1.207414	-0.746223
Sb	2.296637	3.16105	0.746223
Sb	0	3.907273	-0.746223
Sb	-2.296637	3.16105	0.746223
Sb	-3.716037	1.207414	-0.746223
Sb	1.4194	-1.953636	-3.16105
Sb	2.296637	0.746223	-3.16105

Sb	0	2.414827	-3.16105
Sb	-2.296637	0.746223	-3.16105
Sb	-1.4194	-1.953636	-3.16105

$Sb_{20} \subset He$

He	0	0	0
Sb	-2.296638	-0.746223	3.16105
Sb	0	-2.414828	3.16105
Sb	2.296638	-0.746223	3.16105
Sb	1.4194	1.953637	3.16105
Sb	-1.4194	1.953637	3.16105
Sb	0	-3.907273	0.746223
Sb	-2.296638	-3.16105	-0.746223
Sb	-3.716038	-1.207414	0.746223
Sb	2.296638	-3.16105	-0.746223
Sb	3.716038	-1.207414	0.746223
Sb	3.716038	1.207414	-0.746223
Sb	2.296638	3.16105	0.746223
Sb	0	3.907273	-0.746223
Sb	-2.296638	3.16105	0.746223
Sb	-3.716038	1.207414	-0.746223
Sb	1.4194	-1.953637	-3.16105
Sb	2.296638	0.746223	-3.16105
Sb	0	2.414828	-3.16105
Sb	-2.296638	0.746223	-3.16105
Sb	-1.4194	-1.953637	-3.16105

$Sb_{20} \subset Ne$

Ne	0	0	0
Sb	-2.296638	-0.746223	3.16105

Sb	0	-2.414828	3.16105
Sb	2.296638	-0.746223	3.16105
Sb	1.4194	1.953637	3.16105
Sb	-1.4194	1.953637	3.16105
Sb	0	-3.907273	0.746223
Sb	-2.296638	-3.16105	-0.746223
Sb	-3.716038	-1.207414	0.746223
Sb	2.296638	-3.16105	-0.746223
Sb	3.716038	-1.207414	0.746223
Sb	3.716038	1.207414	-0.746223
Sb	2.296638	3.16105	0.746223
Sb	0	3.907273	-0.746223
Sb	-2.296638	3.16105	0.746223
Sb	-3.716038	1.207414	-0.746223
Sb	1.4194	-1.953637	-3.16105
Sb	2.296638	0.746223	-3.16105
Sb	0	2.414828	-3.16105
Sb	-2.296638	0.746223	-3.16105
Sb	-1.4194	-1.953637	-3.16105

$Sb_{20} \subset Ar$

Ar	0	0	0
Sb	-2.301249	-0.747721	3.167397
Sb	0	-2.419676	3.167397
Sb	2.301249	-0.747721	3.167397
Sb	1.42225	1.957559	3.167397
Sb	-1.42225	1.957559	3.167397
Sb	0	-3.915118	0.747721
Sb	-2.301249	-3.167397	-0.747721
Sb	-3.723499	-1.209838	0.747721

Sb	2.301249	-3.167397	-0.747721
Sb	3.723499	-1.209838	0.747721
Sb	3.723499	1.209838	-0.747721
Sb	2.301249	3.167397	0.747721
Sb	0	3.915118	-0.747721
Sb	-2.301249	3.167397	0.747721
Sb	-3.723499	1.209838	-0.747721
Sb	1.42225	-1.957559	-3.167397
Sb	2.301249	0.747721	-3.167397
Sb	0	2.419676	-3.167397
Sb	-2.301249	0.747721	-3.167397
Sb	-1.42225	-1.957559	-3.167397

$Sb_{20} \subset Kr$

Kr	0	0	0
Sb	-2.30532	-0.749044	3.173001
Sb	0	-2.423957	3.173001
Sb	2.30532	-0.749044	3.173001
Sb	1.424766	1.961022	3.173001
Sb	-1.424766	1.961022	3.173001
Sb	0	-3.922045	0.749044
Sb	-2.30532	-3.173001	-0.749044
Sb	-3.730086	-1.211979	0.749044
Sb	2.30532	-3.173001	-0.749044
Sb	3.730086	-1.211979	0.749044
Sb	3.730086	1.211979	-0.749044
Sb	2.30532	3.173001	0.749044
Sb	0	3.922045	-0.749044
Sb	-2.30532	3.173001	0.749044
Sb	-3.730086	1.211979	-0.749044

Sb	1.424766	-1.961022	-3.173001
Sb	2.30532	0.749044	-3.173001
Sb	0	2.423957	-3.173001
Sb	-2.30532	0.749044	-3.173001
Sb	-1.424766	-1.961022	-3.173001

$Sb_{20} \subset Xe$

Xe	0	0	0
Sb	-2.312611	-0.751413	3.183036
Sb	0	-2.431623	3.183036
Sb	2.312611	-0.751413	3.183036
Sb	1.429272	1.967224	3.183036
Sb	-1.429272	1.967224	3.183036
Sb	0	-3.934449	0.751413
Sb	-2.312611	-3.183036	-0.751413
Sb	-3.741883	-1.215812	0.751413
Sb	2.312611	-3.183036	-0.751413
Sb	3.741883	-1.215812	0.751413
Sb	3.741883	1.215812	-0.751413
Sb	2.312611	3.183036	0.751413
Sb	0	3.934449	-0.751413
Sb	-2.312611	3.183036	0.751413
Sb	-3.741883	1.215812	-0.751413
Sb	1.429272	-1.967224	-3.183036
Sb	2.312611	0.751413	-3.183036
Sb	0	2.431623	-3.183036
Sb	-2.312611	0.751413	-3.183036
Sb	-1.429272	-1.967224	-3.183036

$Sb_{20} \subset Rn$

Rn	0	0	0
Sb	-2.315974	-0.752505	3.187664
Sb	0	-2.435159	3.187664
Sb	2.315974	-0.752505	3.187664
Sb	1.43135	1.970085	3.187664
Sb	-1.43135	1.970085	3.187664
Sb	0	-3.94017	0.752505
Sb	-2.315974	-3.187664	-0.752505
Sb	-3.747324	-1.217579	0.752505
Sb	2.315974	-3.187664	-0.752505
Sb	3.747324	-1.217579	0.752505
Sb	3.747324	1.217579	-0.752505
Sb	2.315974	3.187664	0.752505
Sb	0	3.94017	-0.752505
Sb	-2.315974	3.187664	0.752505
Sb	-3.747324	1.217579	-0.752505
Sb	1.43135	-1.970085	-3.187664
Sb	2.315974	0.752505	-3.187664
Sb	0	2.435159	-3.187664
Sb	-2.315974	0.752505	-3.187664
Sb	-1.43135	-1.970085	-3.187664

Bi_{20}

Bi	-2.414939	-0.784661	3.323879
Bi	0	-2.539217	3.323879
Bi	2.414939	-0.784661	3.323879
Bi	1.492515	2.05427	3.323879
Bi	-1.492515	2.05427	3.323879
Bi	0	-4.10854	0.784661
Bi	-2.414939	-3.323879	-0.784661
Bi	-3.907454	-1.269609	0.784661

Bi	2.414939	-3.323879	-0.784661
Bi	3.907454	-1.269609	0.784661
Bi	3.907454	1.269609	-0.784661
Bi	2.414939	3.323879	0.784661
Bi	0	4.10854	-0.784661
Bi	-2.414939	3.323879	0.784661
Bi	-3.907454	1.269609	-0.784661
Bi	1.492515	-2.05427	-3.323879
Bi	2.414939	0.784661	-3.323879
Bi	0	2.539217	-3.323879
Bi	-2.414939	0.784661	-3.323879
Bi	-1.492515	-2.05427	-3.323879

$Bi_{20} \subset He$

He	0	0	0
Bi	-2.415105	-0.784715	3.324106
Bi	0	-2.539391	3.324106
Bi	2.415105	-0.784715	3.324106
Bi	1.492617	2.054411	3.324106
Bi	-1.492617	2.054411	3.324106
Bi	0	-4.108821	0.784715
Bi	-2.415105	-3.324106	-0.784715
Bi	-3.907721	-1.269696	0.784715
Bi	2.415105	-3.324106	-0.784715
Bi	3.907721	-1.269696	0.784715
Bi	3.907721	1.269696	-0.784715
Bi	2.415105	3.324106	0.784715
Bi	0	4.108821	-0.784715
Bi	-2.415105	3.324106	0.784715
Bi	-3.907721	1.269696	-0.784715

Bi	1.492617	-2.054411	-3.324106
Bi	2.415105	0.784715	-3.324106
Bi	0	2.539391	-3.324106
Bi	-2.415105	0.784715	-3.324106
Bi	-1.492617	-2.054411	-3.324106

$Bi_{20} \subset Ne$

Ne	0	0	0
Bi	-2.414444	-0.7845	3.323197
Bi	0	-2.538697	3.323197
Bi	2.414444	-0.7845	3.323197
Bi	1.492208	2.053849	3.323197
Bi	-1.492208	2.053849	3.323197
Bi	0	-4.107697	0.7845
Bi	-2.414444	-3.323197	-0.7845
Bi	-3.906652	-1.269348	0.7845
Bi	2.414444	-3.323197	-0.7845
Bi	3.906652	-1.269348	0.7845
Bi	3.906652	1.269348	-0.7845
Bi	2.414444	3.323197	0.7845
Bi	0	4.107697	-0.7845
Bi	-2.414444	3.323197	0.7845
Bi	-3.906652	1.269348	-0.7845
Bi	1.492208	-2.053849	-3.323197
Bi	2.414444	0.7845	-3.323197
Bi	0	2.538697	-3.323197
Bi	-2.414444	0.7845	-3.323197
Bi	-1.492208	-2.053849	-3.323197

$Bi_{20} \subset Ar$

Ar	0	0	0
Bi	-2.415875	-0.784965	3.325167
Bi	0	-2.540201	3.325167
Bi	2.415875	-0.784965	3.325167
Bi	1.493093	2.055066	3.325167
Bi	-1.493093	2.055066	3.325167
Bi	0	-4.110132	0.784965
Bi	-2.415875	-3.325167	-0.784965
Bi	-3.908968	-1.270101	0.784965
Bi	2.415875	-3.325167	-0.784965
Bi	3.908968	-1.270101	0.784965
Bi	3.908968	1.270101	-0.784965
Bi	2.415875	3.325167	0.784965
Bi	0	4.110132	-0.784965
Bi	-2.415875	3.325167	0.784965
Bi	-3.908968	1.270101	-0.784965
Bi	1.493093	-2.055066	-3.325167
Bi	2.415875	0.784965	-3.325167
Bi	0	2.540201	-3.325167
Bi	-2.415875	0.784965	-3.325167
Bi	-1.493093	-2.055066	-3.325167

$Bi_{20} \subset Kr$

Kr	0	0	0
Bi	-2.420488	-0.786464	3.331516
Bi	0	-2.545052	3.331516
Bi	2.420488	-0.786464	3.331516
Bi	1.495944	2.05899	3.331516
Bi	-1.495944	2.05899	3.331516
Bi	0	-4.11798	0.786464

Bi	-2.420488	-3.331516	-0.786464
Bi	-3.916432	-1.272526	0.786464
Bi	2.420488	-3.331516	-0.786464
Bi	3.916432	-1.272526	0.786464
Bi	3.916432	1.272526	-0.786464
Bi	2.420488	3.331516	0.786464
Bi	0	4.11798	-0.786464
Bi	-2.420488	3.331516	0.786464
Bi	-3.916432	1.272526	-0.786464
Bi	1.495944	-2.05899	-3.331516
Bi	2.420488	0.786464	-3.331516
Bi	0	2.545052	-3.331516
Bi	-2.420488	0.786464	-3.331516
Bi	-1.495944	-2.05899	-3.331516

$Bi_{20} \subset Xe$

Xe	0	0	0
Bi	-2.427353	-0.788695	3.340965
Bi	0	-2.55227	3.340965
Bi	2.427353	-0.788695	3.340965
Bi	1.500187	2.06483	3.340965
Bi	-1.500187	2.06483	3.340965
Bi	0	-4.12966	0.788695
Bi	-2.427353	-3.340965	-0.788695
Bi	-3.92754	-1.276135	0.788695
Bi	2.427353	-3.340965	-0.788695
Bi	3.92754	-1.276135	0.788695
Bi	3.92754	1.276135	-0.788695
Bi	2.427353	3.340965	0.788695
Bi	0	4.12966	-0.788695

Bi	-2.427353	3.340965	0.788695
Bi	-3.92754	1.276135	-0.788695
Bi	1.500187	-2.06483	-3.340965
Bi	2.427353	0.788695	-3.340965
Bi	0	2.55227	-3.340965
Bi	-2.427353	0.788695	-3.340965
Bi	-1.500187	-2.06483	-3.340965

$Bi_{20} \subset Rn$

Rn	0	0	0
Bi	-2.432004	-0.790206	3.347367
Bi	0	-2.557161	3.347367
Bi	2.432004	-0.790206	3.347367
Bi	1.503061	2.068786	3.347367
Bi	-1.503061	2.068786	3.347367
Bi	0	-4.137573	0.790206
Bi	-2.432004	-3.347367	-0.790206
Bi	-3.935066	-1.27858	0.790206
Bi	2.432004	-3.347367	-0.790206
Bi	3.935066	-1.27858	0.790206
Bi	3.935066	1.27858	-0.790206
Bi	2.432004	3.347367	0.790206
Bi	0	4.137573	-0.790206
Bi	-2.432004	3.347367	0.790206
Bi	-3.935066	1.27858	-0.790206
Bi	1.503061	-2.068786	-3.347367
Bi	2.432004	0.790206	-3.347367
Bi	0	2.557161	-3.347367
Bi	-2.432004	0.790206	-3.347367
Bi	-1.503061	-2.068786	-3.347367

Table S2. Number of imaginary frequencies calculated at the ωB97XD/ def2-svp/svpfit theory level for $X_{20}H_{20}$ host

	C	Si	Ge	Sn	Pb
Noble gas	Nimag	Nimag	Nimag	Nimag	Nimag
empty	0	0	0	0	8[&]
He	0	3[§]	0	0	8[&]
Ne	0	0	0	0	8[&]
Ar	0	0	0	0	8[&]
Kr	0	0	0	0	8[&]
Xe	0	0	0	0	8[&]
Rn	0	0	0	0	8[&]

[§]Noble gas is moving inside the cage's cavity for comparability noted altered to lower symmetry

[&]The $Pb_{20}H_{20}$ skeleton undergoes small vibrations, elongating them and recalculating without symmetry leading to slightly distorted pentagonal dodecahedral structures. For comparability the reported systems with higher symmetry were selected.

Table S3. Number of imaginary frequencies calculated at the ωB97XD/ def2-svp/svpfit theory level for X_{20} host

	N	P	As	Sb	Bi
Noble gas	Nimag	Nimag	Nimag	Nimag	Nimag
empty	0	0	0	0	0
He	0	0	3[§]	0	0
Ne	0	0	0	0	0
Ar	0	0	0	0	0
Kr	13[&]	0	0	0	0
Xe	17[&]	0	0	0	0
Rn	23[&]	0	0	0	0

[§] Noble gas is moving inside the cage's cavity for comparability noted altered to lower symmetry

[&] Noble gases of a relatively bigger radii are too small for the selected hosts cavity

Table S4. Number of imaginary frequencies calculated on different theory levels for the $Pb_{20}H_{20}$ and Bi_{20} hosts.

Noble gas	$Pb_{20}H_{20}{}^{a}$	$Bi_{20}{}^{a}$	$Pb_{20}H_{20}{}^{b}$	$Bi_{20}{}^{b}$
empty	8	0	8	0
He	8	0	8	0
Ne	8	0	8	0

Ar	8	0	8	0
Kr	8	0	8	0
Xe	8	0	8	0
Rn	8	0	8	0

[a]APFD/def2-svp/svpfit
[b]B3LYP- GD3BJ /def2-svp/svpfit

Table S5. Gaussian keywords input templates for the performed calculations.

#P RwB97XD/def2svp/svpfit opt freq=noraman

#P APFD/def2svp/svpfit opt freq=noraman

#P RB3LYP/ def2svp/svpfit EmpiricalDispersion=GD3BJ opt freq=noraman

#P RwB97XD/def2svp/def2-tzvp

#P test RwB97XD/def2tzvp Counterpoise=2

0,1 0,1 0,1

Ng(Fragment=1)

Cage(Fragment=2)

….

#P RwB97XD/def2tzvp POP=chelp

#P RwB97XD/def2svp/def2-tzvp

#P RAPFD/def2tzvp

#P RB3LYP/def2tzvp EmpiricalDispersion=GD3BJ

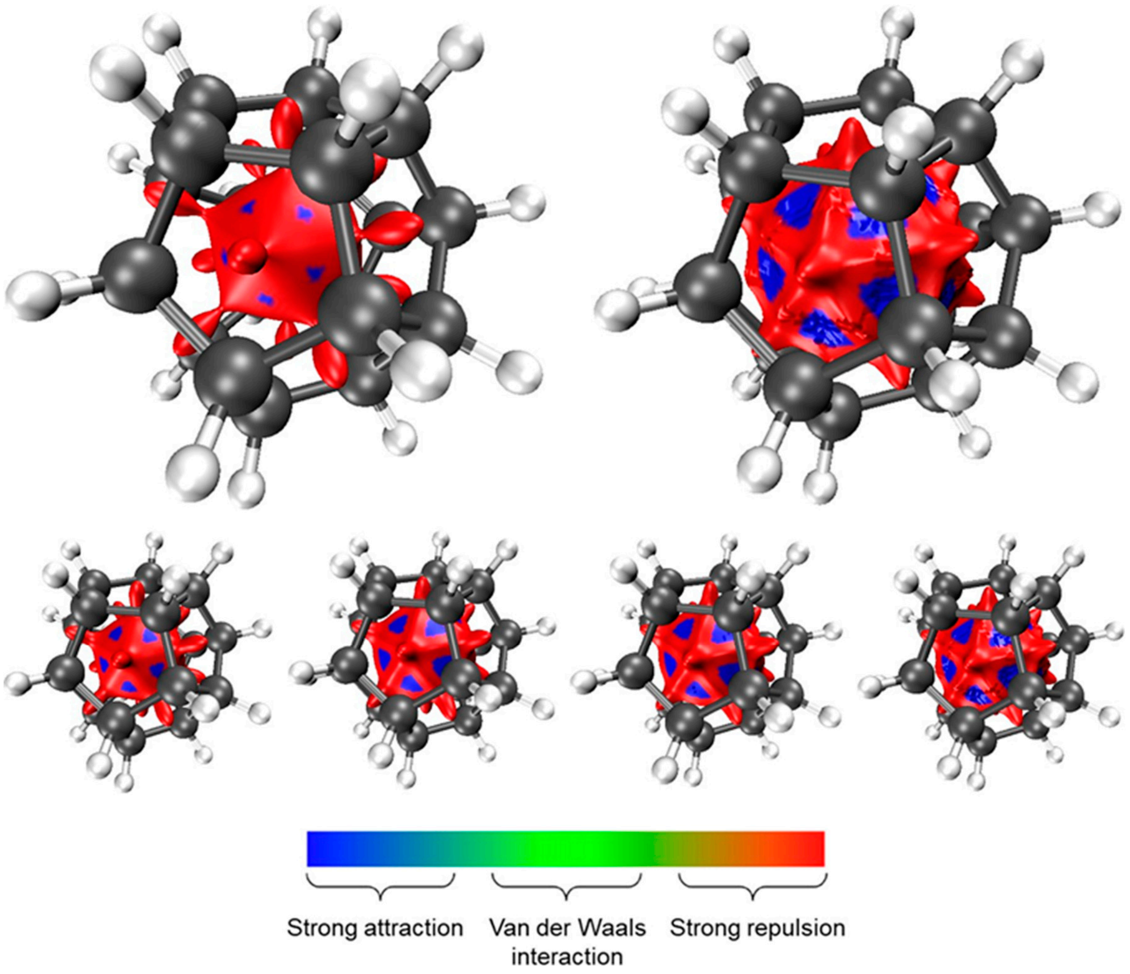

Figure S1. Structure of $C_{20}H_{20}$ with displayed non-covalent interactions according to the color bar (isoval = 0.18). Top row: He and Rn. Bottom row: Ne, Ar, Kr and Xe.

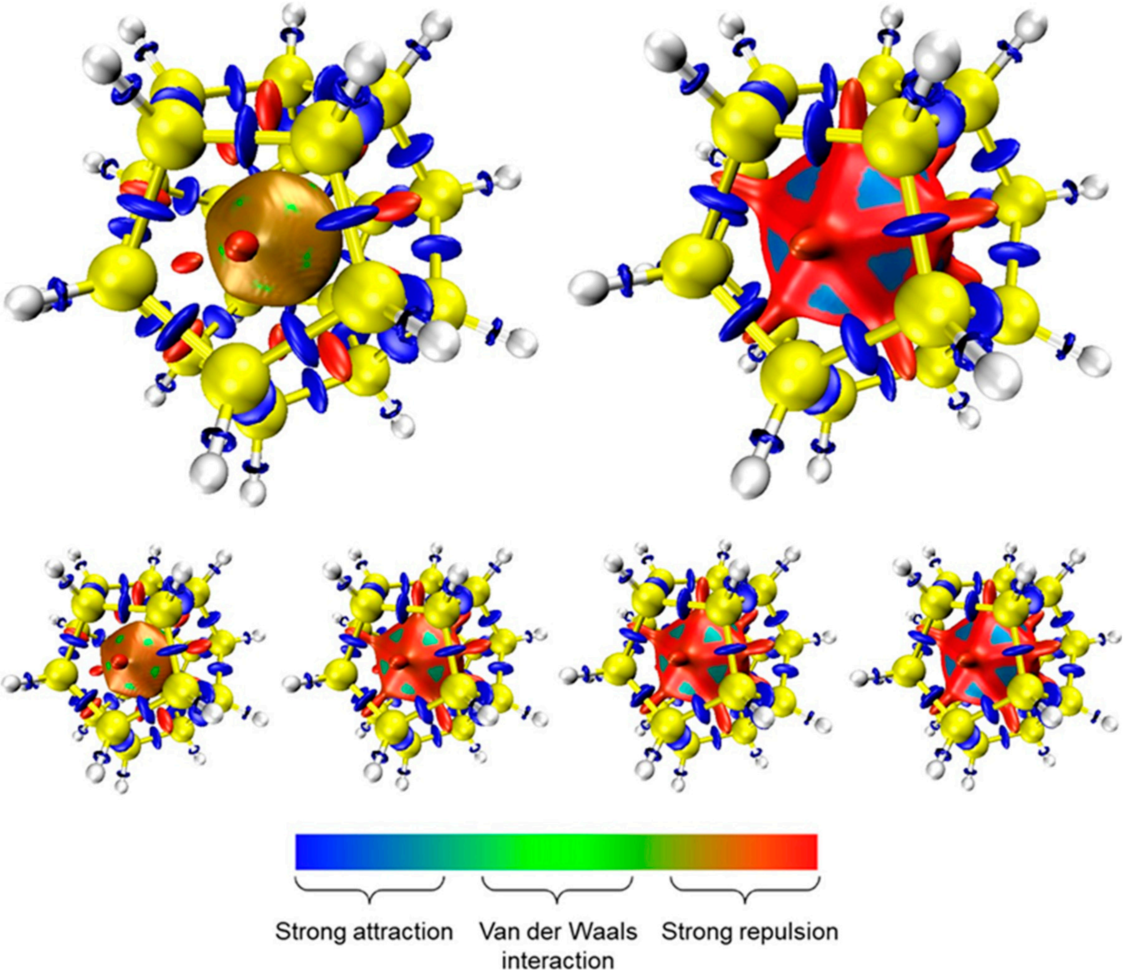

Figure S2. Structure of $Si_{20}H_{20}$ with displayed non-covalent interactions according to the color bar (isoval = 0.22). Top row: He and Rn. Bottom row: Ne, Ar, Kr and Xe.

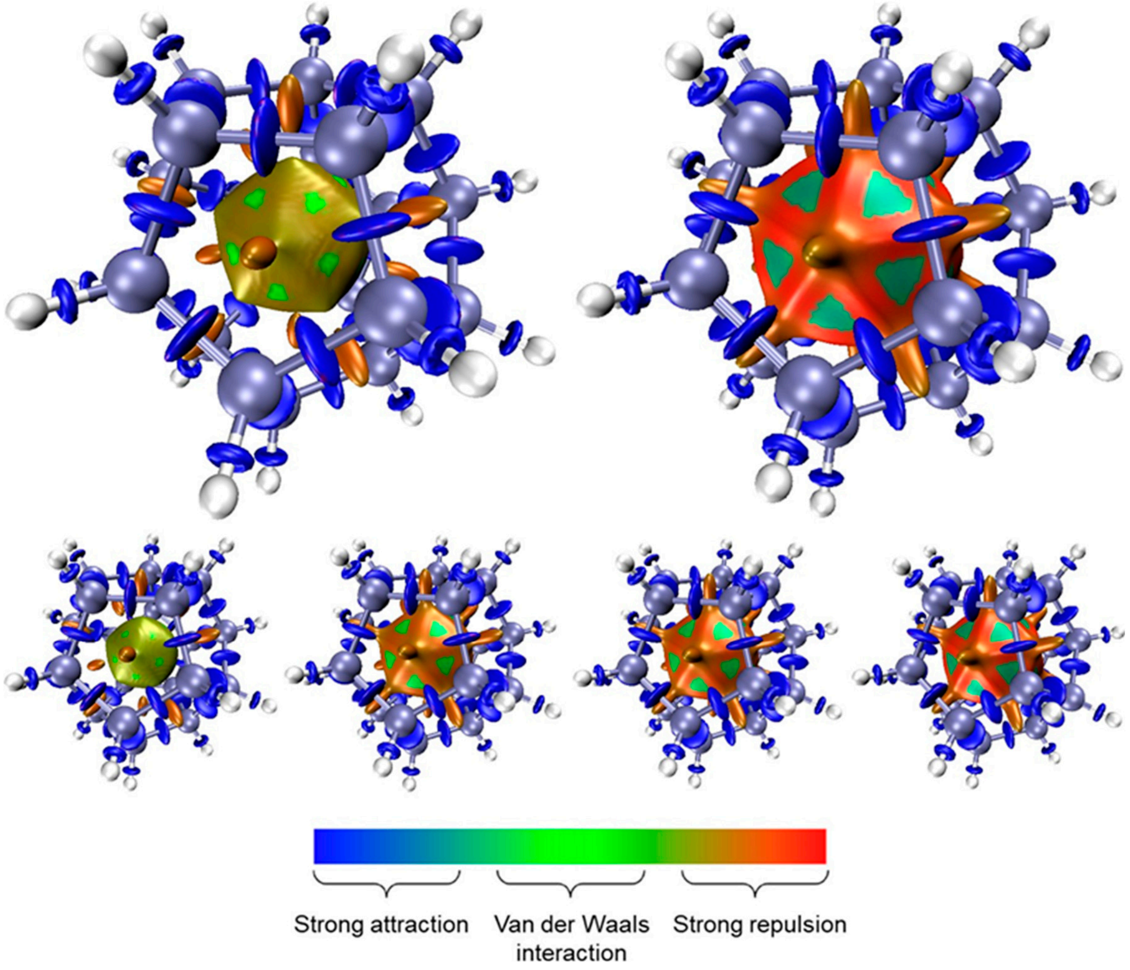

Figure S3. Structure of $Ge_{20}H_{20}$ with displayed non-covalent interactions according to the color bar (isoval = 0.3). Top row: Ne and Rn. Bottom row: He, Ar, Kr and Xe.

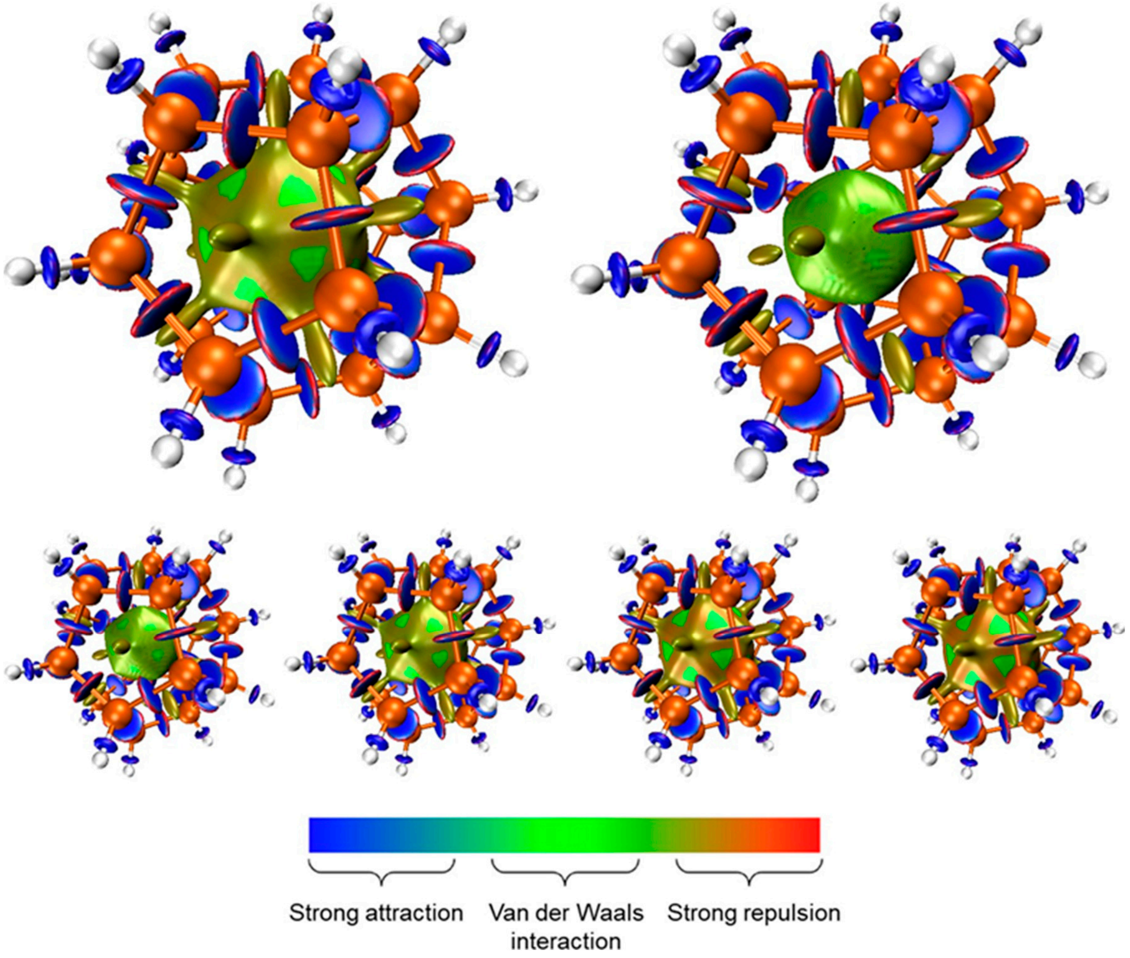

Figure S4. Structure of $Sn_{20}H_{20}$ with displayed non-covalent interactions according to the color bar (isoval = 0.35). Top row: Kr and He. Bottom row: Ne, Ar, Xe and Rn.

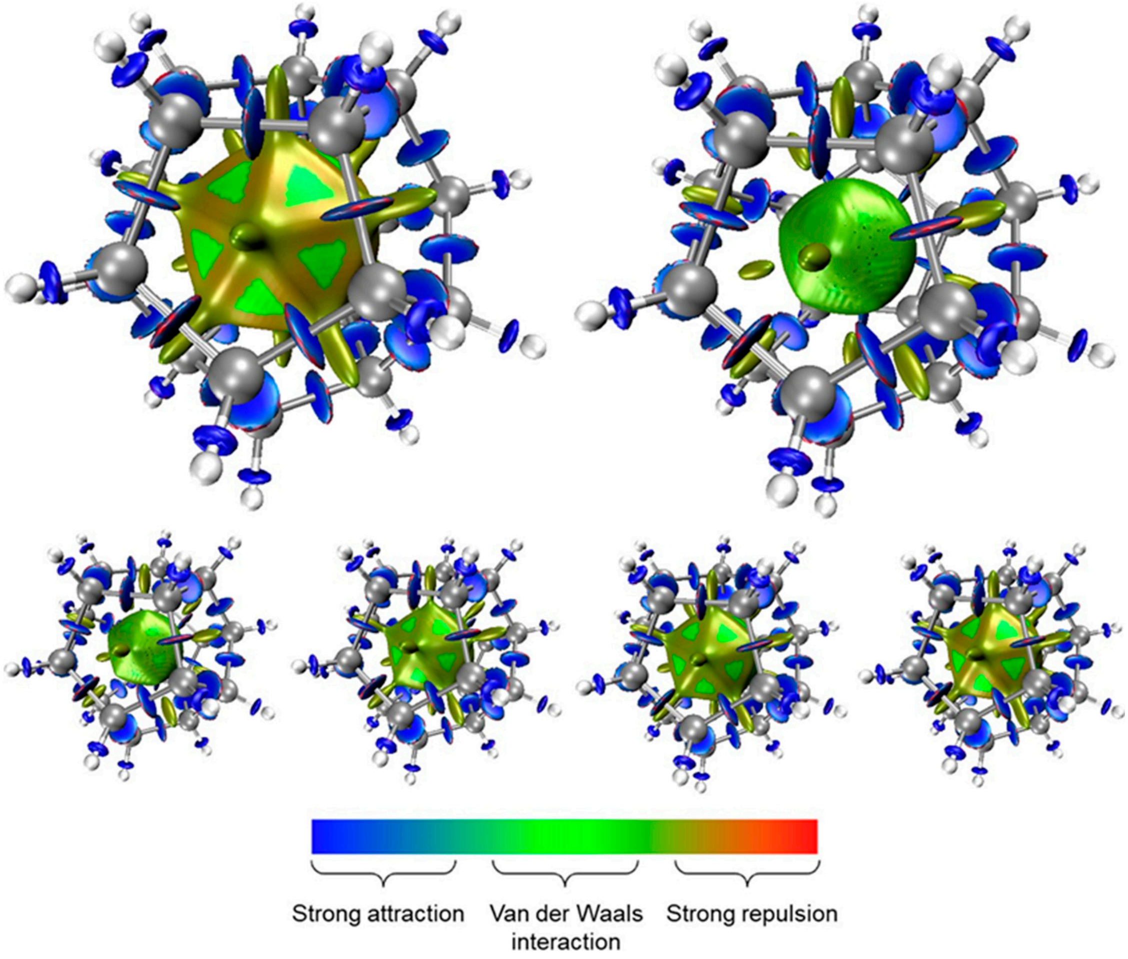

Figure S5. Structure of $Pb_{20}H_{20}$ with displayed non-covalent interactions according to the color bar (isoval = 0.35). Top row: Rn and He. Bottom row: Ne, Ar, Kr and Xe.

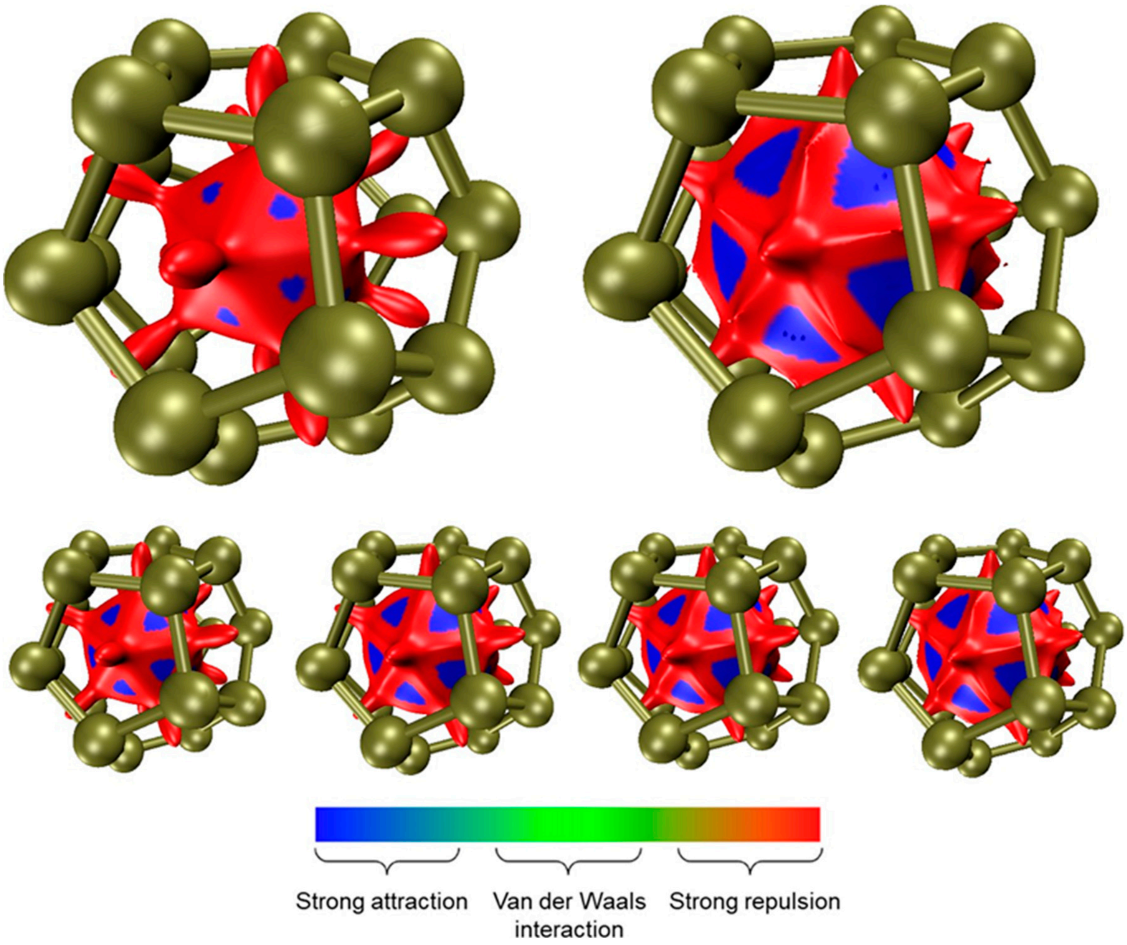

Figure S6. Structure of N_{20} with displayed non-covalent interactions according to the color bar (isoval = 0.25). Top row: He and Rn. Bottom row: Ne, Ar, Kr and Xe.

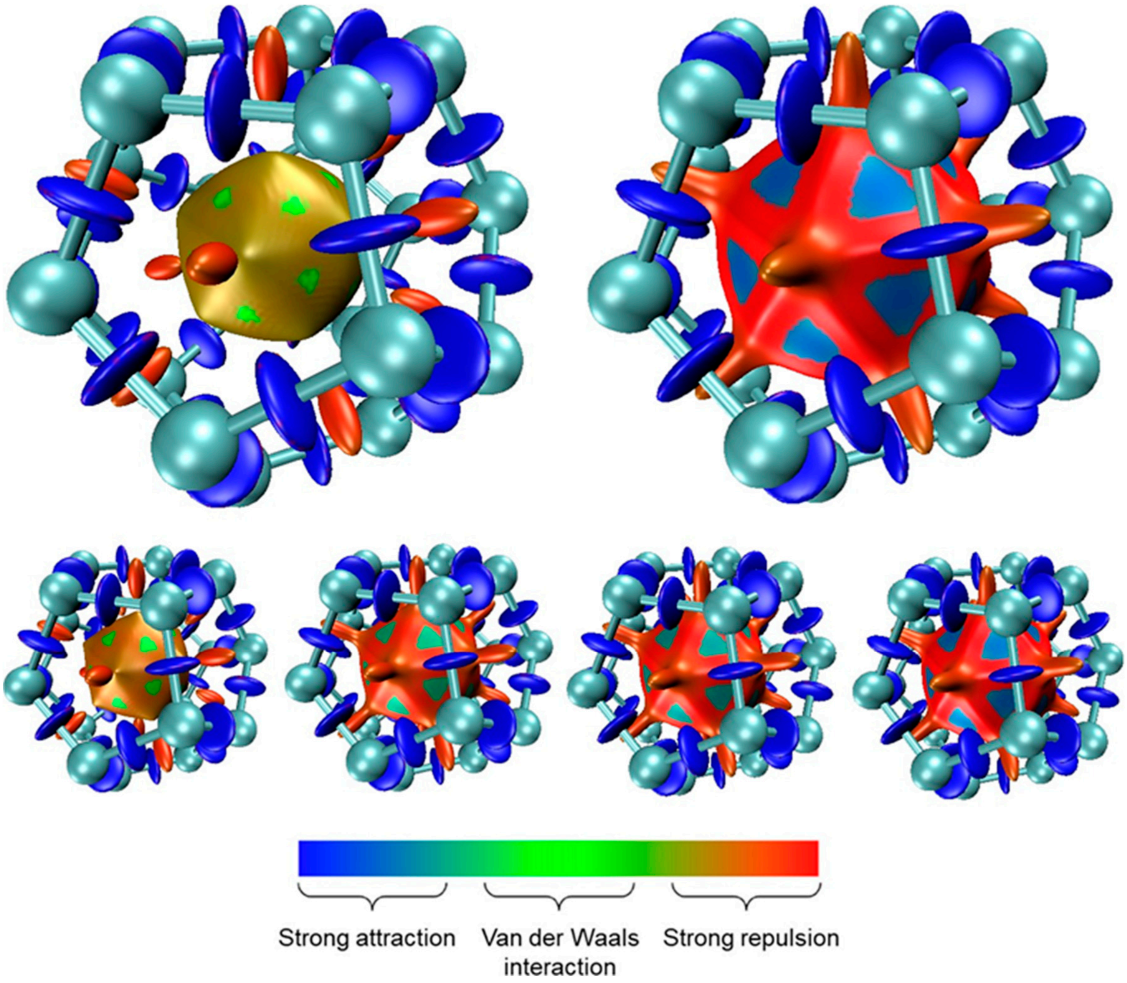

Figure S7. Structure of P_{20} with displayed non-covalent interactions according to the color bar (isoval = 0.3). Top row: He and Rn. Bottom row: Ne, Ar, Kr and Xe.

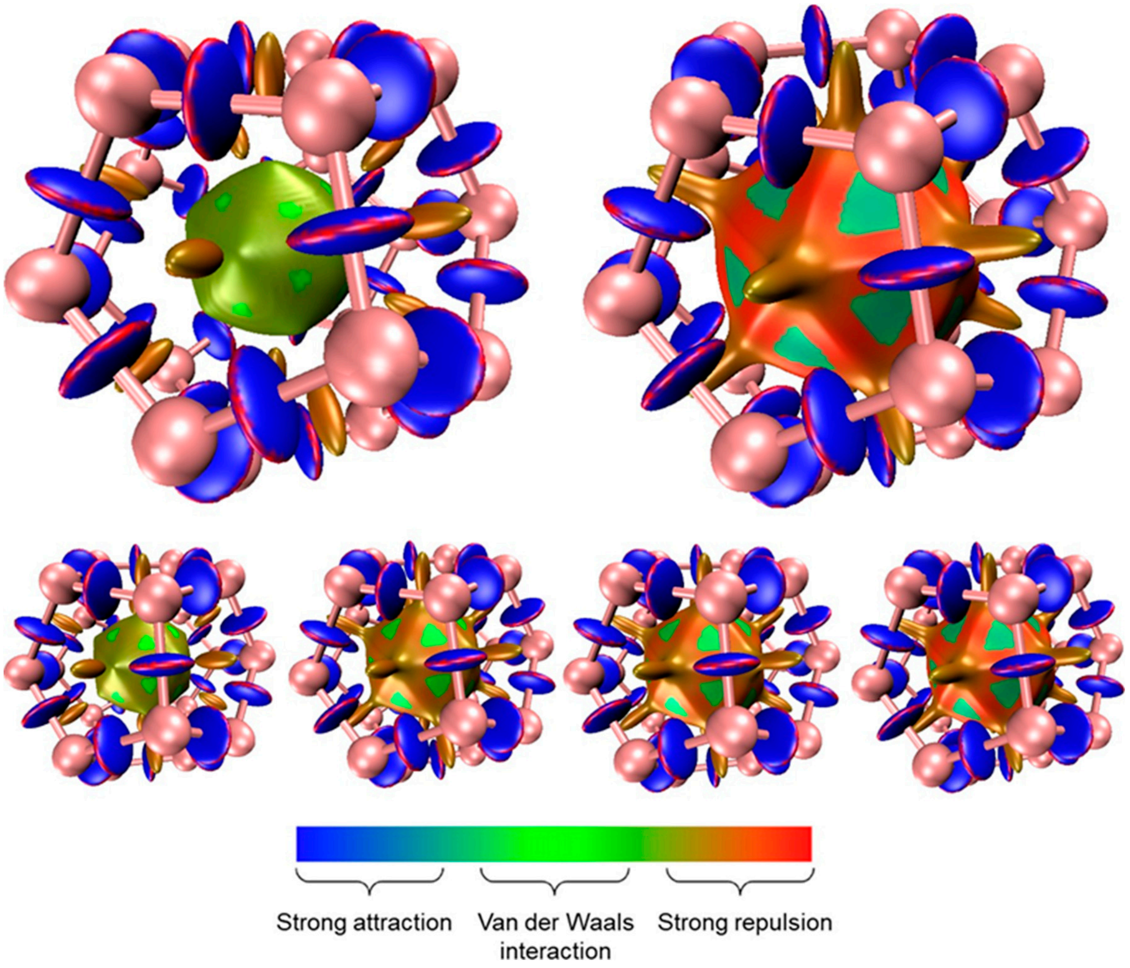

Figure S8. Structure of As_{20} with displayed non-covalent interactions according to the color bar (isoval = 0.35). Top row: He and Rn. Bottom row: Ne, Ar, Kr and Xe.

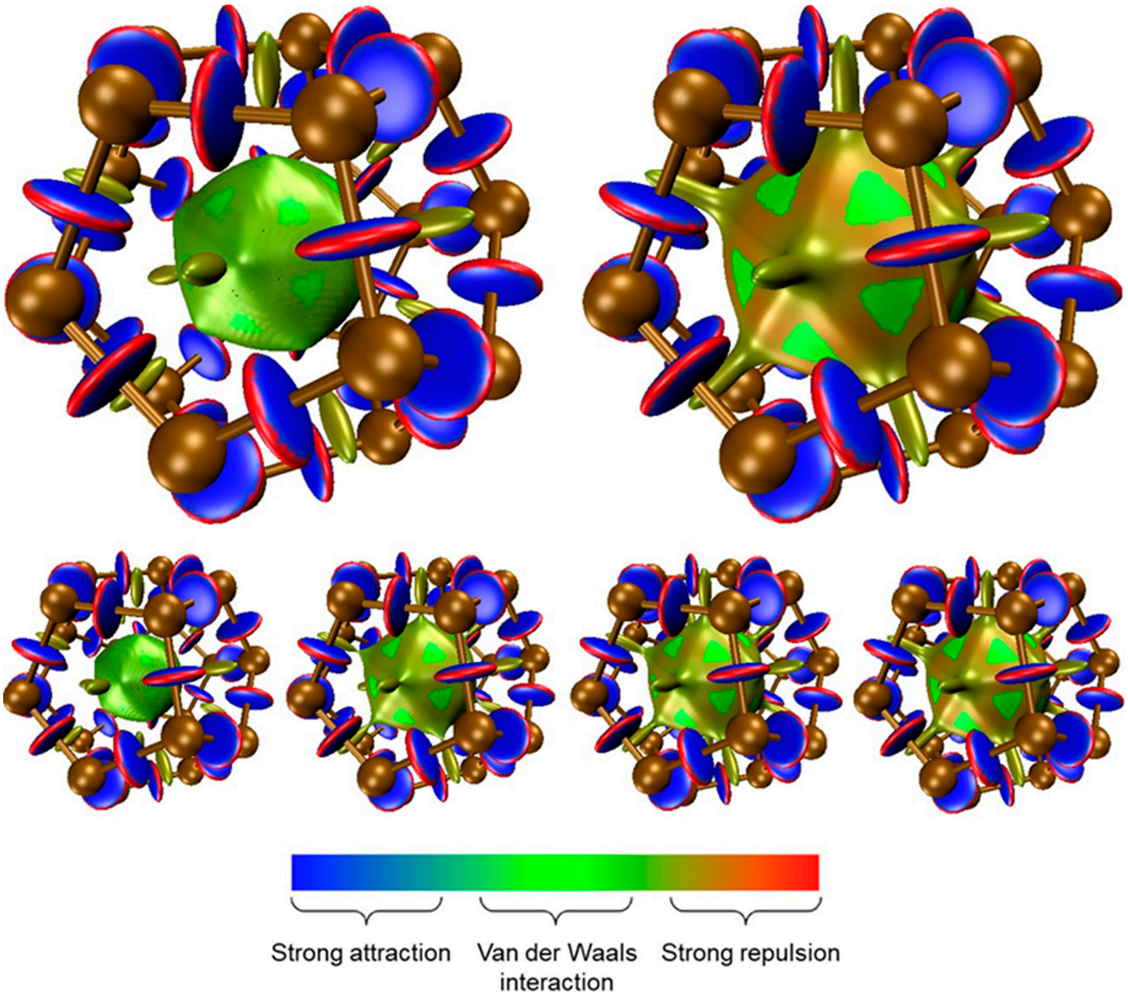

Figure S9. Structure of Sb_{20} with displayed non-covalent interactions according to the color bar (isoval = 0.37). Top row: Ne and Rn. Bottom row: He, Ar, Kr and Xe.
